# Genetically Modified Plants in Agriculture

**DOI:** 10.3390/biology15120923

**Published:** 2026-06-12

**Authors:** Anna A. Ogienko, Elina S. Surkova, Evgeniya S. Omelina

**Affiliations:** 1 Institute of Molecular and Cellular Biology, Siberian Branch, Russian Academy of Sciences (IMCB SB RAS), 630090 Novosibirsk, Russia; 2 Novosibirsk State University, 630090 Novosibirsk, Russia

**Keywords:** genetically modified (GM) plants, *Agrobacterium*, transformation, transgenes, agriculture, breeding

## Abstract

Genetically modified (GM) plants have revolutionized agriculture for more than three decades. The production of a GM plants is a complex, multi-stage process. Several key methods are available for generating GM plants. The choice of transformation method depends on the type of plant (dicotyledonous or monocotyledonous), the objective (large-scale production versus studying a specific gene in particular cells or tissues), and whether stable or transient transformation is desired. Following successful transformation, the next step is the regeneration of a whole plant from a single cell in tissue culture, which is a labor-intensive and time-consuming process. Currently, numerous genes that confer desirable traits have been identified. These traits include stress tolerance, herbicide and pest resistance, and improved consumer qualities (such as flavor, appearance, shelf life, and nutritional value). In this review, we describe the main methods for producing GM plants and provide examples of trait genes utilized in agricultural biotechnology. Despite the fact that GM plants represent one of the most significant biotechnological advances, they also remain among the most contentious issues in contemporary food safety and agricultural policy. Here, we discuss the advantages and disadvantages of using GM plants for humans.

## Simple Summary

Genetically modified plants contain an insertion, deletion or edited version of genes of interest within their genomes. A trait gene may originate from an unrelated plant or a different species and can confer specific characteristics (e.g., pest resistance, drought tolerance, salinity tolerance, or resistance to extreme temperatures) or enable the production of proteins with industrial or pharmaceutical value. The first genetically modified plant, tobacco, was produced in the early 1980s. Since then, many plants with a variety of desired traits have been developed. This review outlines the main methods for producing genetically modified plants, lists trait genes employed in agricultural biotechnology, and presents the advantages and disadvantages of utilizing genetically modified plants in agriculture.

## 1. Introduction

Plant gene technology involves transferring genes with known functional traits, such as high yield potential, stress resistance, disease tolerance, and enhanced nutritional profiles, into target plants or editing or removing specific genes from the plant genome using advanced scientific methodologies. This process results in the acquisition of novel functional properties while preserving the original genetic foundations of the plant [1,2]. The first genetically modified (GM) plant was produced in the early 1980s by incorporating the *neomycin phosphotransferase II* (*nptII*) gene, which confers resistance to the antibiotics kanamycin and neomycin, into tobacco [3]. Since that time, a multitude of GM plants with various desirable traits have been developed. There are several basic methods for producing GM plants; however, the majority have been generated using the biolistic method or through the use of *Agrobacterium*. Transgenes may originate from an unrelated plant or from an entirely different species and can endow the plant with specific traits or enable the production of proteins with industrial and pharmaceutical value [2].

In this review, we outline the main methods for producing GM plants, compare traditional and molecular plant breeding, and provide some examples of trait genes used in agriculture. Additionally, we discuss the advantages and disadvantages of using GM plants in the context of their impact on human health.

## 2. Transformation Methods

**Figure 1:**
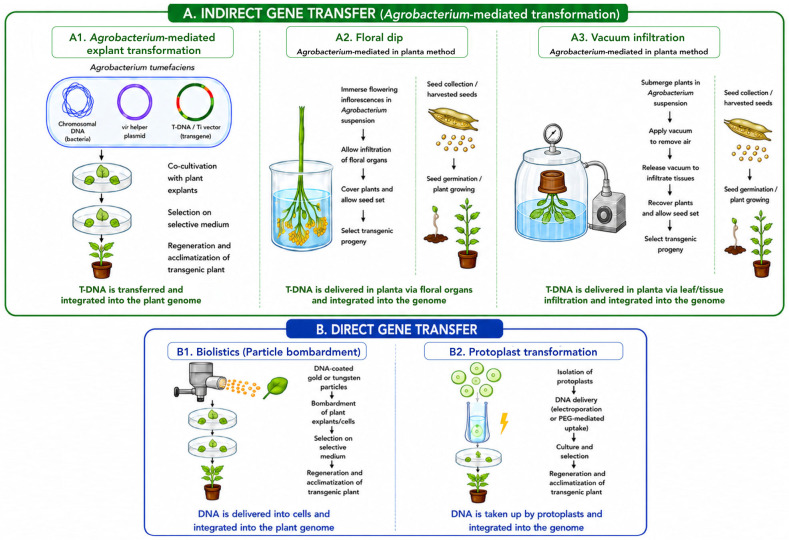
Indirect and direct DNA-transfer methods in plant transformation.

The hallmark of GM technology is its ability to transfer genes across species, thereby enabling the introduction of desirable traits that may not be naturally present in the target organism. These genes can be sourced from the same or closely related species, or from different species entirely, which expands the genetic resources available for crop improvement [4] compared to those available through traditional techniques [5]. The choice of methodology for obtaining a GM plant is determined by several factors: (1) whether the plant belongs to the class of dicotyledons or monocotyledons. For most dicotyledons, *Agrobacterium*-mediated transformation is preferred, whereas, in the case of monocotyledons, biolistics is more commonly employed; (2) the type of target tissue. Various tissues and organs can be subject to transformation, including leaf pieces (discs), immature embryos, callus cells, and pollen; and (3) the research objectives. To study transient gene expression, simple delivery methods for individual cells are suitable. To obtain a stable inheritable line, gene integration into the genome followed by the regeneration of the plant is required. Each method has its own advantages and limitations [6], and the selection of the optimal approach is crucial for successfully creating a transgenic plant with the desired traits—such as resistance to herbicides, pests, or abiotic stresses, or improvements in nutritional quality. All methods of plant transformation can be divided into two main types: indirect (also known as vector-mediated gene transfer) and direct gene transfer of foreign DNA into the plant cell (Figure 1).

### 2.1. Vector-Mediated Gene Transfer

#### 2.1.1. “*Agrobacterium*” Method

This method is based on the utilization of *Agrobacterium* and the ability of these bacteria to transfer their genes into the plant genome. The species most commonly used for transformation is *A. tumefaciens*, which contains the tumor-inducing (Ti) plasmid responsible for causing crown gall disease. A key component of the Ti plasmid is the region of transfer DNA (T-DNA), which encodes the biosynthesis of opines and phytohormones. The three oncogenes (involved in the biosynthesis of opines, cytokinins, and auxins) located within the T-DNA are the primary causes of tumor formation in plants. Plant transformation employs modified agrobacteria that lack tumor-promoting genes or opine-synthesis genes in their genome. A vector is created based on the Ti plasmid, from which these native genes are removed to prevent harm to the plant. In their place, the desired trait gene, a selection marker, and optionally a reporter gene for visual confirmation of successful transformation are inserted.

There are two main types of vectors used for *Agrobacterium*-mediated plant transformation: cointegrate vectors and binary vectors [7]. The strategy based on cointegrate vectors is not widely employed today due to the complex engineering required for these vectors. A more commonly utilized and convenient system for *Agrobacterium*-mediated transformation is the T-binary vector system, which comprises two components: the T-binary vector and the *vir* helper plasmid (Figure 1A) [8,9,10]. The T-binary vector contains T-DNA border repeats derived from the Ti plasmid as well as the genes of interest. It can replicate independently in both *E*. *coli* and agrobacteria, separate from the bacterial chromosome. After the production and purification of the T-binary vector from *E*. *coli*, it is transformed into agrobacteria, such as strains LBA4404, EHA105, AGL-1, or GV3101 [11,12,13]. These strains harbor the *vir* helper plasmid, which is a disarmed Ti plasmid devoid of tumor-related genes, responsible for the synthesis of Vir proteins [8,14]. Agrobacteria containing the T-binary vector are cultivated in a nutrient medium supplemented with appropriate antibiotics to ensure an adequate number of cells for plant infection. For the transformation process, plant tissues or explants exhibiting a wounded surface are employed. During the co-cultivation of these damaged plant parts with a suspension of agrobacteria, plant cells release a specific phenolic compound (acetosyringone), which activates *vir* genes in the agrobacteria [15,16,17,18]. The products of the *vir* genes, along with certain proteins, facilitate the excision and transfer of single-stranded T-DNA from the T-binary vector into the host plant cell [19]. Within the nucleus, the T-DNA is integrated into the plant genome with the assistance of Vir proteins. Subsequently, the explants are transferred to a nutrient medium containing antibiotics to eliminate the agrobacteria and to select for transformed cells.

The subsequent step, the regeneration of a whole, fertile plant from a transformed cell in tissue culture, represents a critical phase [20,21]. This developmental process predominantly occurs through two distinct pathways: indirect organogenesis and somatic embryogenesis. In the case of indirect organogenesis, dedifferentiation is initiated by a precise balance of exogenous auxins and cytokinins on the surface of the wounded explant, resulting in the formation of a callus—an unorganized mass of cells. Subsequently, shoot apical meristems are induced on a selective medium characterized by a high cytokinin-to-auxin ratio, followed by root induction on an auxin-enriched medium [22]. During somatic embryogenesis, transformed cells undergo a fate transition and dedifferentiation to acquire embryogenic competence. These cells then develop into bipolar somatic embryos, either completely bypassing the callus phase or transitioning through a brief, transient callus stage [23].

In vitro regeneration remains the most labor-intensive step and acts as the primary bottleneck (recalcitrance) in generating transgenic lines (Table 1) [24]. This limitation is particularly pronounced in monocotyledonous crops, where regeneration efficiencies remain strictly dependent on specific, hard-to-obtain explants, severely restricting high-throughput genetic engineering and genome editing [25].

At present, numerous protocols feature various modifications of the plant transformation process using *Agrobacterium* [26,27]. These modifications include the optimization of the nutrient medium composition, bacterial strains, types of explants, and the use of hormones and antibiotics. Moreover, superbinary vectors have emerged as an improved version of binary vectors, carrying additional *vir* genes responsible for the supervirulence phenotype of the bacteria [28,29]. The superbinary vector-based system exhibits a remarkably high frequency of transformation, which is particularly valuable for recalcitrant plants such as cereals [28]. In addition, in planta transformation protocols have emerged, significantly increasing transformation efficiency and allowing for the successful modification of both dicotyledonous and monocotyledonous plants, including major cereal crops such as wheat, rice, and maize [30,31,32,33,34].

The two most common *Agrobacterium*-mediated in planta methods are floral dip and vacuum infiltration (Figure 1A) [35]. The floral dip method involves immersing flowers in an *Agrobacterium* suspension [36,37]. The resulting transgenic seeds are collected directly from the plant. While this approach is quick and practical, it exhibits low transformation efficiency and is primarily suitable for dicotyledonous plants [36,38,39,40,41] and some monocot plants [42,43].

Vacuum infiltration, where plant tissue or whole plants are submerged in a liquid suspension of *A*. *tumefaciens* and subjected to reduced pressure followed by rapid repressurisation, is a common method for introducing bacteria into plant tissue (Figure 1A) [33,44]. The vacuum infiltration method allows for the production of both transgenic seeds and transgenic vegetative parts of plants. This approach has long been employed to increase transformation efficiency in numerous plants, including both dicotyledonous and monocotyledonous species, as it enhances the penetration of agrobacteria into the layers of plant tissue [45,46]. Furthermore, the vacuum infiltration method can be complemented by sonication, resulting in even higher transformation efficiency [47,48,49,50,51].

#### 2.1.2. Methods Based on Plant Virus Vectors

Plant virus vectors are employed as tools for the effective and precise delivery of genetic material into plants [52,53]. RNA and DNA viruses differ fundamentally in their infection mechanisms and are therefore utilized to deliver distinct types of molecular cargo [54]. The most common plant viruses contain single-stranded RNA (ssRNA) and are classified into two types: positive-strand ((+)ssRNA) and negative-strand ((−)ssRNA) viruses [54].

The majority of (+)ssRNA viruses can only accommodate insertions of a few hundred nucleotides (Table 1) [55,56,57]. Due to their limited cargo capacity and genetic instability, (+)ssRNA viruses are unable to deliver long foreign sequences (for instance, the Cas9 open reading frame [58]), but they are well suited for smaller cargoes such as guide RNAs.

(−)ssRNA viruses exhibit greater genome stability and higher cargo capacity compared with (+)ssRNA viruses [58,59,60,61]. They are capable of accommodating an entire CRISPR/Cas cassette, thereby eliminating the need for a Cas nuclease-expressing transgenic recipient plant. However, (−)ssRNA viruses are excluded from the meristem and cannot deliver editing reagents into germ cells [62]. Consequently, edited somatic cells must be regenerated into whole plants that can transmit the genetic modifications to their progeny [54].

Plant DNA viruses are less abundant compared with plant RNA viruses. Among them are the single-stranded DNA (ssDNA) viruses belonging to the families *Geminiviridae* [63,64] and *Nanoviridae* [65], as well as the double-stranded DNA (dsDNA) plant pararetroviruses of the family *Caulimoviridae* [66,67].

Geminiviruses have facilitated the development of virus vector systems capable of delivering long donor DNA fragments and achieving high copy numbers [68,69]. Additionally, Geminiviruses have been utilized as vectors for virus-induced gene silencing (VIGS), which degrades transcripts of endogenous plant genes without altering the genes themselves or necessitating stable genetic transformation (Table 1). Furthermore, Geminivirus-based vectors offer a robust platform for delivering genome editing reagents. Virus-induced genome editing (VIGE) is a technique that employs engineered plant viruses to transport CRISPR/Cas components into plant cells. Typically, the viral genome is encoded within the T-DNA borders of a binary vector, and the virus is assembled upon delivery to the plant cell. Due to its high replication rate, the CRISPR/Cas components are expressed at elevated levels [70].

Members of the family *Caulimoviridae* have primarily been utilized as sources of regulatory elements [71]. The most notable example is the 35S promoter from Cauliflower mosaic virus (CaMV), which remains one of the strongest constitutive promoters available for use in plants [72]. However, their application as replicating vectors is limited by a narrow host range and difficulties associated with the insertion of foreign DNA fragments. The size of exogenous DNA that can be successfully propagated within viral particles is restricted to approximately 250 bp [73,74].

Several techniques are available for delivering viral vectors into plants, including mechanical inoculation [75,76,77], foliar spraying [78,79], needle-laden injection into the stem or petiole [78], agroinfiltration [55,80], and the biolistic method [54,78].

### 2.2. Direct Gene Transfer

#### 2.2.1. Biolistic Method

The ‘Gene Gun’, also known as the particle bombardment method or biolistics, is one of the most widely used techniques for plant transformation [81,82]. This approach is particularly useful for plant species that are difficult to transform using *Agrobacterium*-mediated methods, including many cereals and legumes [6]. The name of the technique derives from the process by which cells are ‘shot’ with genetic material. In practice, the procedure begins by mixing the DNA of interest with tiny particles made of gold or tungsten. These metal particles carry a positive charge, which binds them to the negatively charged DNA. Once coated, the DNA-metal particles are loaded into a gene gun or biolistic device. A pressurized gas, typically helium, then propels a microcarrier bearing the DNA-coated particles towards a stopping screen. As the gas pressure increases, the microcarrier forces the DNA-metal particles through the screen, allowing them to penetrate the cell membranes and deliver the DNA constructs directly into the nucleus of target cells placed in a Petri dish (Figure 1B) [83]. Following delivery, the DNA detaches from the metal particles and may be integrated into the plant’s chromosomal DNA by endogenous recombination mechanisms.

Biolistics is effective for transforming both dicotyledonous and monocotyledonous plants (Table 1). Similar to *Agrobacterium*-mediated transformation, it can achieve both stable and transient gene expression, as well as the simultaneous delivery of large numbers of different genetic elements [84]. The technique is relatively non-toxic and can be used to introduce DNA into almost any type of tissue, including immature and mature embryos, shoot apical meristems, leaves, roots, and others [85]. However, this method has several disadvantages, which are detailed in Table 1.

#### 2.2.2. Transformation of Protoplasts

Protoplast transformation is a technique used to introduce foreign DNA into plant cells that lack a cell wall, known as protoplasts or naked cells. This approach is commonly employed to study gene function, determine protein localization, or perform CRISPR/Cas9 genome editing. It is particularly useful for transient gene expression, allowing the analysis of multiple genes within a short timeframe (Table 1).

In practice, protoplasts are first generated through enzymatic digestion that breaks down the cell wall. Notably, protoplasts largely retain the cellular identity and differentiated characteristics of their original source cells [86]. Consequently, the isolation procedure must be tailored to each species, organ, or tissue [87]. Following isolation, direct DNA delivery into individual plant cells is achieved using either polyethylene glycol (PEG) or electroporation (Figure 1B) [88,89,90]. PEG-mediated transformation is an effective and widely adopted method for directly delivering DNA into protoplasts. In the presence of calcium ions, PEG induces reversible membrane fusion and promotes the uptake of exogenous DNA via endocytosis [91]. Electroporation-based protoplast transformation uses electrical pulses to generate transient pores in the plant cell membrane, thereby facilitating the entry of foreign DNA [92]. After transformation, protoplasts are selectively cultured and can be used for subsequent regeneration steps [93].

#### 2.2.3. Microinjection

The microinjection technique represents a direct physical strategy for delivering DNA into selected cells [94,95]. It employs a fine glass needle operated under microscopic observation, allowing precise injection without harming the target cells. This method is labor-intensive and requires both expensive equipment and considerable technical skill. In practice, the target cell is held steady beneath a microscope using two micromanipulators. One of these functions as a holding pipette to secure the cell in place, while the other is a microcapillary tube filled with a small volume of DNA solution, intended to pierce either the plasma membrane or the nuclear envelope. Through this process, DNA is introduced into the cytoplasm or nucleus of plant cells or protoplasts using a microcapillary pipette. Following the completion of gene transfer, the transformed cells are cultured and eventually regenerated into whole plants. This technique can be applied both to individual cells and to protoplasts.

#### 2.2.4. Nanotechnologies

Nanotechnologies facilitate precise genetic transformation in plants by circumventing the rigid plant cell wall through the use of nanoparticles, which are ultrafine particles with diameters of less than 100 nm. Various types of nanoparticles, including carbon dots, as well as single-walled and multi-walled carbon nanotubes, gold nanoparticles (nanospheres, nanorods, nanoclusters), silicon-based carriers, magnetic nanoparticles, chitosan nanoparticles, and bio-inspired carriers such as liposomes and vesicles, are capable of transporting different types of cargo, including drugs, proteins, and nucleic acids, into plant cells [96,97,98]. The plant transformation approach using nanoparticles exploits the similar electrical charge near the cell membrane to overcome the barrier of the plant cell wall, thereby introducing various cargoes into plant tissues to achieve either transient or stable transformation [99]. Additionally, nanoparticles have been successfully employed for organelle-targeted gene delivery [100].

## 3. Plant Breeding: Traditional Versus Molecular Approaches

Using the aforementioned techniques, genetic engineering is carried out, which involves the precise, direct modification of an organism’s DNA using recombinant DNA technology or gene-editing tools like CRISPR. It inserts, removes, or alters specific genes to introduce entirely new and highly targeted traits. Genetic engineering, alongside marker-assisted selection (MAS) and genomic selection, is a component of molecular breeding [101].

**Figure 2:**
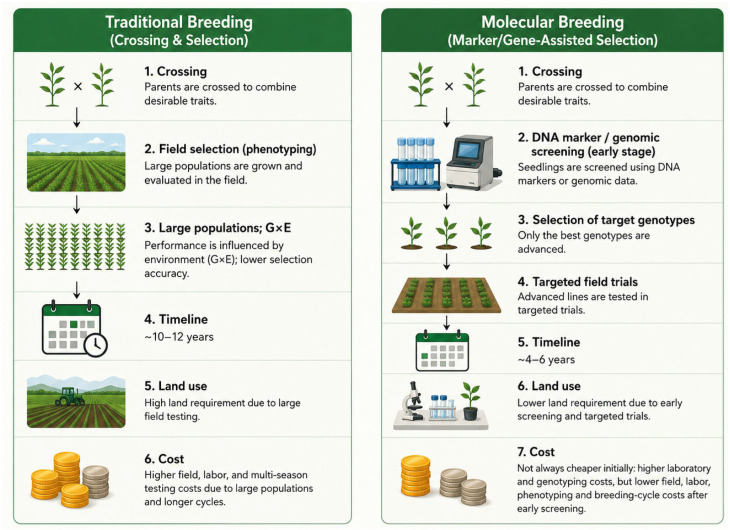
Comparison between traditional and molecular breeding frameworks.

Molecular breeding overcomes several limitations of traditional breeding by combining marker- and genome-based selection with targeted genetic modification approaches. DNA markers, genome sequencing, and genomic selection enable breeders to identify desirable genotypes at the molecular level before full phenotypic expression, whereas transgenesis and genome editing allow for the direct modification of genetic variation. By shifting the focus from visible phenotypes to molecular markers, genomic loci, and genotype-phenotype associations, molecular tools can reduce the time, space, and resources required to identify complex, recessive, or environmentally sensitive traits [102]. This distinction between phenotype-led and genotype-informed breeding is summarized in Figure 2, which contrasts the conventional workflow of crossing, phenotypic selection, and field-testing with molecular approaches based on marker detection, genomic prediction, and targeted genetic modification.

**Figure 3:**
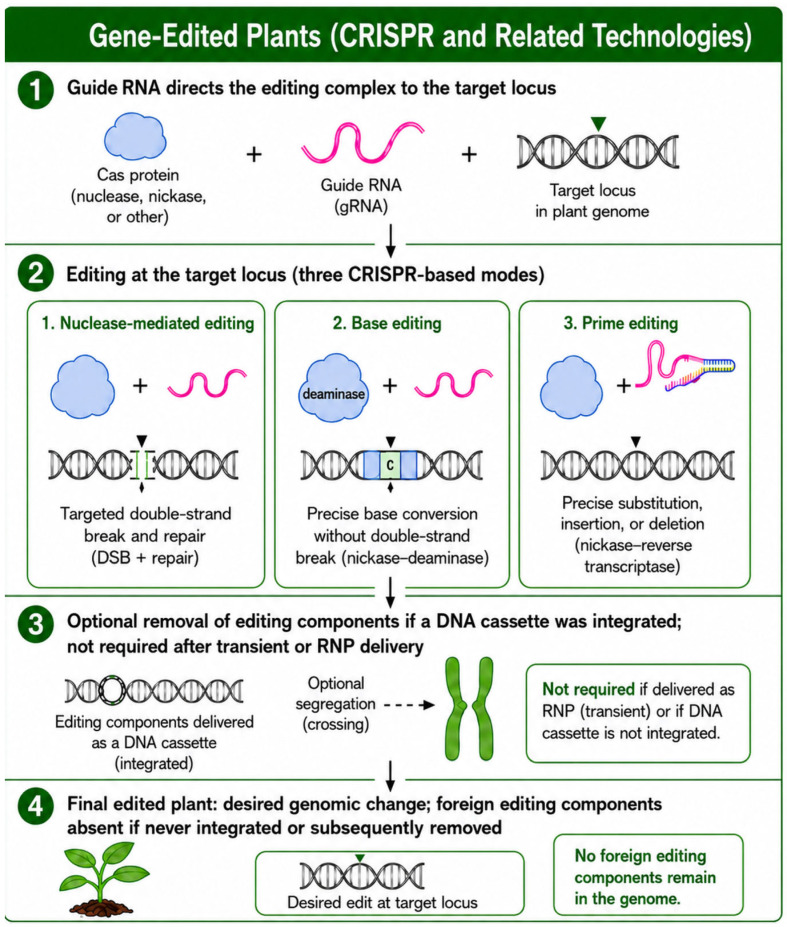
CRISPR/Cas genome editing.

Gene editing, particularly CRISPR/Cas-based systems, extends molecular breeding beyond selection by enabling targeted DNA modification. Unlike conventional GM technologies, which typically introduce transgenes into the host genome, CRISPR/Cas systems can introduce programmable, site-specific changes (Figure 3). In some instances, CRISPR-edited plants can be generated without retaining foreign DNA [5].

Molecular breeding is also experiencing a temporal shift. Earlier approaches primarily focused on single-nucleotide polymorphisms (SNPs), quantitative trait loci (QTL) mapping, and MAS, whereas modern applications increasingly trend towards broader genome engineering. Recent developments include programmed structural variation, such as large insertions, duplications, inversions, and transposable-element-based genome reshaping [103]. Furthermore, base editing expands CRISPR-based breeding by enabling targeted nucleotide conversion while avoiding the formation of double-strand breaks, making it beneficial for the precise modification of agriculturally relevant alleles [104]. Additionally, CRISPR-based epigenome editing further extends this approach from DNA-sequence alteration to regulatory control. In *Arabidopsis*, directed manipulation of the H3K4me3 chromatin mark has been employed to influence endogenous gene expression, resistance-related responses, and recombination in low-recombining genomic regions [105].

Ultimately, molecular breeding should not be regarded as a substitute for traditional methods. Although molecular tools expedite genetic advancement and improve precision, field validation remains essential, as the ultimate agronomic performance is significantly influenced by environmental conditions and genotype × environment (G×E) interactions (Figure 2). Therefore, the ideal modern breeding paradigm is an integrated one: conventional breeding supplies agronomic and field-testing frameworks, while molecular biology, genome editing, AI-assisted prediction, and tissue-culture-free delivery strategies provide mechanisms for more precise and accelerated selection or modification.

## 4. Trait Genes

Currently, numerous plants have been artificially created by humans and possess specific transgenes that confer particular traits. In this section, we consider a number of genes used to endow agricultural crops with various beneficial properties, such as herbicide resistance, enhanced biomass, resistance to pests and diseases, improved consumer qualities, stress response, and resistance to adverse environmental factors.

### 4.1. Genes Providing Herbicide Resistance

Herbicides have a complex and far-reaching impact on plant genes [106]. They can induce random chromosomal changes and mutations or serve as a powerful force of natural selection, eliminating susceptible individuals and promoting the accumulation of plants possessing protective mutations within populations [107]. There are two distinct types of herbicide resistance mechanisms: target-site resistance (TSR) and non-target-site resistance (NTSR) [107,108]. TSR involves mutations within the genes that code for herbicide target proteins [109]. Most of these mutations are non-synonymous substitutions that lead to an amino acid change, thereby preventing the herbicide from binding to the protein without disrupting the enzyme’s normal function [110]. TSR may also result from increased expression of the target gene, resulting in the production of more enzyme than can be effectively inhibited by standard herbicide application rates [111,112].

NTSR is a complex and rapidly evolving mechanism that enables weeds to survive following herbicide treatment. Whereas TSR arises from mutations in a specific target protein, NTSR can involve a large number of genes and is achieved by reducing the quantity of active herbicide through sequestration, decreased herbicide absorption, translocation, and enhanced metabolism [113,114]. This form of resistance is particularly concerning because it often confers cross-resistance to herbicides with different modes of action [115]. NTSR presents a significant challenge for agriculture. This resistance mechanism typically evolves as a consequence of prolonged herbicide application, during which various resistance alleles accumulate within a population. Due to its polygenic basis and its ability to provide cross-resistance to new or even yet-to-be-utilized herbicides, weeds possessing NTSR are considerably more difficult to manage than those with TSR.

Examples of genes that confer herbicide resistance are presented in Table 2.

### 4.2. Genes Responsible for Increasing Biomass 

Genetic engineering offers opportunities to significantly enhance the productivity of agricultural crops. Researchers are developing transgenic varieties that exhibit faster growth, produce greater biomass, and possess improved tolerance to environmental stress. These advancements are achieved through the introduction of genes that regulate key physiological processes in plants, ranging from the control of photosynthetic efficiency to the modulation of plant hormonal systems. Such genes include the following: *RPS6K2* [158], *fto* [159], *CYP85A3* [160], *D11*-*2A* [161], *PTR6* [162], *NFYA*-*B1* [163], *NLP7* [164], and others. In Table 3, we present some of the genes that enhance biomass.

### 4.3. Genes for Enhancing Resistance to Pests and Diseases

Improving the resistance of crops to pests and diseases is achieved through the use of specific genetic approaches, notably resistance genes, susceptibility gene editing, and RNA interference (RNAi) [186,187]. Key strategies include the introduction of resistance genes, which enable plants to recognize pathogens, and the application of CRISPR/Cas9 to disrupt susceptibility genes that pathogens rely upon [188,189]. The most widely used resistance genes are *Cry* transgenes derived from the soil bacterium *Bacillus thuringiensis* (Bt) [190]. Other genes employed for the protection of plants against pests and diseases include *Vip* [191], *CpTI* [192], *bgn13.1* [193], *NPR1* [194]. Some of the genes are presented in more detail in Table 4.

### 4.4. Genes Improving Consumer Properties

Transgenes that enhance the consumer properties of a plant encompass genes responsible for improving flavor, appearance, nutritional value, texture, and shelf life. For instance, in tomatoes, numerous genes that regulate fruit coloration, morphology, flavor, and nutritional value have been identified (reviewed in [218]). In rice, modifications have been made to both amino acid and protein composition [219,220], as well as micronutrient content [221] (reviewed in [222]). In *Zea mays* L. *saccharata*, the combination of the *sh2* and *se1* genes, or the *su1* and *se1* genes, yields superior hybrids with improved taste, shelf stability, and consumer appeal (reviewed in [223]). In Table 5, we present some genes that enhance the consumer properties of crops.

### 4.5. Genes Providing Resistance to Adverse Environmental Conditions

Rather than introducing a single stress resistance gene, scientists often utilize regulatory genes that function as “master switches” for plant stress tolerance. Such genes encode proteins that do not directly produce protective compounds but instead activate entire cascades of genes responsible for responding to drought, salinity, and extreme temperatures, both high and low. Examples of these genes include *DREB1* [231] and *HSP* [232], which trigger comprehensive defense systems already present within the plant. This approach is highly effective against a range of combined stresses. Other stress response genes include *ABI1*, *HAB1*, and *GSTU17*, which regulate the abscisic acid signaling pathway, thereby enhancing tolerance to drought and salinity [233]; the lipocalin *TIL* and *CHL* genes, which are essential for resistance to abiotic stress and for survival [234]; dehydrins (DHNs), which are activated in vegetative tissues of the plant and confer increased tolerance to drought and salt stress [235]; *eIF2a*, which is crucial for plant survival under conditions of macronutrient starvation [236]; *GATA16*, which improves cold tolerance at the seedling stage in rice [237] and others (see Table 6).

## 5. Global Status of GM Plants in the World

**Figure 4:**
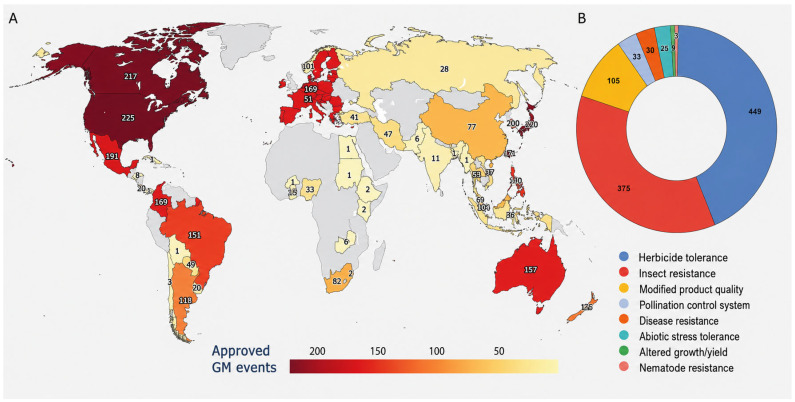
Distribution of approved GM crops. (**A**) Number of approved GM crop events by country or region according to the ISAAA GM Approval Database, accessed in May 2026. “Approved” indicates regulatory authorization for at least one use, such as cultivation, import, food/feed use, or commercialization, while a “GM event” refers to a specific GM plant line generated by a particular transformation event. Values should not be interpreted as the number of cultivars, plant species, or hectares under cultivation. (**B**) Distribution of approved GM crop events by commercial trait category according to the ISAAA GM Approval Database, accessed in May 2026. Event counts represent regulatory approval entries for specific GM events. Values should not be interpreted as numbers of commercial cultivars or hectares under cultivation. As stacked events may contain multiple traits, categories are not mutually exclusive.

Earlier estimates from 2019 reported approximately 190 million hectares of GM crops cultivated across 29 countries, with soybean, maize, cotton, and canola being the predominant crops [5,249]. The adoption landscape had expanded by 2024: from 1996 to 2024, 73 countries had integrated GM crops through either cultivation or imports (Figure 4). In 2024 alone, more than 20 biotech/GM crops were cultivated across 31 countries, according to the ISAAA GM Approval Database (https://www.isaaa.org/gmapprovaldatabase/default.asp (accessed on 28 May 2026)).

## 6. Impact of GM Crops on Human Health

Each country has its own risk assessment protocol, which must be executed before a GM crop can be marketed globally. These protocols are designed to minimize risks to human health and prevent the displacement of natural crop varieties [250,251]. Different countries and authorities may employ their own methods to assess risks. These methods typically consider several factors: (1) how genetic modification can alter a plant’s natural compounds or create entirely new ones; (2) whether the new compounds produced by the plant are safe; (3) analyzing the plant’s metabolites; (4) examining how nutrients in the GM plant have changed. The risk assessment of GM crops concerning human health involves evaluating both direct effects (the GM plants themselves) and indirect effects (GM plants cultivated with chemicals).

### 6.1. The Direct Risk

A comprehensive analysis of health-focused studies reveals consistent safety profiles for approved GM crops across multiple evaluation parameters, including acute toxicity, subchronic effects, and nutritional equivalence [250,252]. Direct adverse effects on human health from the consumption of GM crops are now generally regarded as negligible [249]. Numerous studies have been conducted to assess the safety of GM rice, sugarcane, soybean, maize, and papaya in relation to human health. Scientists have investigated the impact of GM plants on health by feeding model animals (primarily rats and mice) with different GM crops over extended periods (ranging from 26 to 90 days, and in rare instances, up to 8 months). The feeding of model animals with GM plants, such as rice [253,254], sugarcane [255], maize [256,257,258,259,260], papaya [261], and soybean [262,263], has shown no mortality and only negligible changes in other biological parameters such as body weight, plasma protein levels, food utilization rate, and others. All GM plants were considered to be as safe as their non-GM counterparts [251]. Even in cases where transgenic soybeans contained three different resistance genes—conferring resistance to glyphosate (*EPSPS*), glufosinate (*pat*), and lepidopteran pests (*cry1Ac*, *cry2Ab2*, and *mVip3Aa*)—no effects were detected [264]. Studies on larger animals, such as pigs and calves fed with Bt maize, showed that Cry proteins were fragmented and diminished in the gastrointestinal tract and were not absorbed into the spleen, liver, or lymph nodes [265]. Moreover, heating maize prior to consumption further reduces exposure, and Cry proteins have not been detected in processed foods [266], confirming safety for human consumption [251]. A comprehensive seven-year feeding study on two generations of cynomolgus macaques found that GM maize with *Cry1Ab*/*Cry2Aj* and *EPSPS* genes had no major effects on gut microbiota composition, structure, or function [266]. This study is the longest GM crop feeding trial in non-human primates, offering key insights into multigenerational effects. Multigenerational reproductive toxicity studies have confirmed safety over long-term exposure. A three-generation rat study of GM maize with *Cry1Ab* and *EPSPS* genes also found no adverse effects on reproduction, offspring development, or multigenerational health [267]. Similarly, two-generation reproductive toxicity studies of *DREB3* GM wheat in rats demonstrated no treatment-related effects on reproductive function, fertility, or offspring viability [268,269].

### 6.2. The Indirect Risk

GM technology is based on resistance to pesticides, allowing farmers to spray them directly on GM crops. Pairing glyphosate with glyphosate-resistant GM crops removed a natural limitation on glyphosate use—glyphosate kills non-GM crops and targeted weeds. Relaxing this constraint has resulted in dramatic increases in glyphosate application intensity [270]. Glyphosate can affect human health through contaminated water, dust blown by the wind, aerial drift, and direct contact by consuming GM plants treated with glyphosate. Several independent studies have demonstrated that residues of glyphosate herbicides and its breakdown product, AMPA, accumulate in glyphosate-resistant plants [271,272]. The seeds of soybeans sprayed with the recommended glyphosate levels for weed control appear to have the highest reported levels of glyphosate of any food sourced from a glyphosate-resistant crop [273]. The impact of glyphosate on human health has been a matter of concern. While few scientists believe that the association of the herbicide with non-Hodgkin’s lymphoma in humans is conclusive, others argue that current safety standards are outdated and need revision to prevent any long-term health effects from crops [274].

Detecting trace levels of glyphosate in human urine confirms systemic exposure [275]. The rise in endocrine and neurological disorders, such as Alzheimer’s, has also been linked to the increasing use of this pesticide in the USA [251,274]. The negative health impacts of glyphosate exposure in Brazil were demonstrated by Dias et al. [276] and Skidmore et al. [277]. They found that glyphosate exposure—driven by the expansion of GM seeds and transported through rivers—led to increased infant mortality and pediatric cancer deaths in Brazil. In the USA, it was shown that the introduction of GM seeds and glyphosate significantly reduced average birth weight and gestational length [270]. Bt crops provide resistance to lepidopteran insect pests, thereby reducing the need for chemical insecticide sprays against these specific pests. Numerous studies have indeed found evidence that the adoption of Bt crops is associated with significant reductions in insecticide use [278,279,280]. Furthermore, Bt maize contains a much lower concentration of highly hazardous mycotoxins than regular maize, making it considerably safer for human consumption [281]. Bt crops also offer direct health benefits to farmers through reduced insecticide exposure during spraying operations [281].

## 7. Discussion

The adoption of GM crops has been rapid and widespread across several major crop-producing countries around the world. Despite a vast number of GM plants known to possess trait genes for improved consumer qualities, pest and disease resistance, and enhanced productivity and immunity (Table 2, Table 3, Table 4 and Table 5), adoption is primarily limited to three GM traits: herbicide tolerance, Bt tolerance, and product quality (Figure 4B), along with a small number of other commercial crops (from [249]). The use of GM plants in agriculture represents one of the important and contentious topics for discussion, as this approach has both significant advantages and serious drawbacks, as well as potential risks.

The advantages include increased yield attributable to specific transgenes. For instance, the presence of the Bt transgene in insect-resistant GM crops enables them to produce their own insecticidal proteins, rendering the plants toxic to insect pests. This reduces the need for chemical insecticides and leads to higher yields [282]. Resistance to viral diseases and adverse conditions, such as drought, also contributes to enhanced productivity. Another benefit of employing transgenic plants is the improvement of consumer qualities in agricultural crops. This might include an extended shelf life for fruit in transgenic tomatoes with delayed ripening or enhancements to nutritional value. One of the most renowned examples is Golden Rice, which is enriched with beta-carotene, a precursor of vitamin A (Table 5). Moreover, the improvement of nutritional value may encompass alterations in the composition of fatty acids, as demonstrated in transgenic soybeans. Additionally, by reducing the amount of machinery required to cultivate fields, carbon dioxide emissions into the atmosphere are decreased. The resilience of cultivated crops to adverse conditions (such as drought and soil salinity) also aids in preventing the expansion of agricultural land, thereby contributing to the preservation of forests. From an economic perspective, GM plants in agriculture offer further advantages. With reduced costs for chemical protection, fuel, and labor, this can result in decreased production costs. Furthermore, the use of GM plants tends to yield more predictable and stable results, as crop yields become less reliant on pests and unfavorable conditions.

However, the use of GM plants also carries certain risks. Firstly, there may be ecological risks. The introduction of glyphosate-resistant GM plants has led to a significant increase in the use of glyphosate, from 0.1 kg per hectare of cropland to over 1.3 kg per hectare in the USA [270]. Meanwhile, in the EU, which has never approved GM seeds, the rate of glyphosate application remains close to the United States’ pre-GM levels, at approximately 0.2 kg per hectare [270]. The situation with herbicides mirrors the current scenario with antibiotics. Uncontrolled application of herbicides ultimately results in the evolution of herbicide-resistant weeds, specifically the emergence of so-called “superweeds”. Consequently, fields are treated with more toxic and dangerous herbicide mixtures, which leads to greater accumulation of herbicides and their breakdown products in GM plants, soil, water, and air.

Additionally, there are concerns that pollen from Bt plants may negatively affect beneficial insects, such as bees, or the soil microbiota. Furthermore, genes from transgenic crops may be transferred to wild relatives or organic crops, as controlling the spread of pollen by wind or insects is nearly impossible.

The application of GM plants also carries potential economic risks. Most often, the seeds of transgenic plants are patented by large corporations, which means that farmers must purchase seeds anew each season. This not only makes farmers dependent on multinational corporations, but also potentially leads to an increase in the so-called social divide, as smallholdings in developing countries often cannot afford expensive seeds and the accompanying herbicides.

There are concerns within society regarding the health risks associated with the consumption of GM plants, particularly concerning the potential allergenicity of such crops if the plant carries a gene for an allergen [283]. For example, a Brazil nut gene added to soybeans to boost nutrition triggered allergic reactions in tests [284].

Discussions also focus on the long-term effects of GM crops. The expansion of monocultures and the proliferation of resistant weeds and pests may diminish or even negate the short-term benefits of reduced insecticide use [285]. The long-term impact of GM crop adoption on species groups, including bees, butterflies, and other insects, remains largely unassessed. These groups may be directly affected by changes in GM crops and pesticide usage, and their abundance and diversity may, in turn, directly influence agricultural production [286]. Additionally, the quantification of the impact of GM crop adoption on global deforestation has yet to be undertaken [249]. Skepticism regarding GM crops persists, even in the absence of scientific evidence demonstrating harm from these products. Consequently, the current global approach is divided. In North and South America (the USA, Canada, Brazil, and Argentina), GM crops (such as soya, maize, rapeseed, and cotton) occupy extensive areas and form the backbone of export agriculture (Figure 4A). Conversely, in the EU, Russia, and various countries in Africa and Asia, stringent regulations are enforced concerning the cultivation of GM plants, with only certain crops permitted for import and processing (primarily for animal feed) or a moratorium in place, largely due to perceived environmental and health concerns.

## 8. Conclusions

Thus, GM plants, on the one hand, provide solutions to many problems related to hunger, deficiencies of specific nutrients in certain regions of the world, and the ability to grow and produce yields under unfavorable environmental conditions. On the other hand, the use of GM plants in agriculture carries certain economic and ecological risks. One of the challenges facing the global community at present is to balance the high potential of GM plants to improve agricultural efficiency with the management of the associated risks.

## Figures and Tables

**Table 1:** Advantages and limitations of DNA-transfer methods in plant transformation.

Method	Main Advantages	Key Limitations
*Agrobacterium*-mediated transformation	requires minimal equipment and relatively straightforward operation; low transgene copy number; few DNA rearrangements and comparatively stable transgene expression	many monocots, legumes and woody species remain recalcitrant, because of restricted host range; plant regeneration is highly time-consuming
plant virus vectors	high-level transient expression without genome integration; suitable for VIGS, VIGE, genome-editing reagent delivery and recombinant protein production	limited cargo capacity and genetic instability of many (+)ssRNA virus vectors; restricted meristem/germ cells delivery in some systems; narrow host range or difficulty carrying foreign DNA fragments in certain DNA virus vectors; small insert capacity of Caulimovirus-based particles
biolistics	applicability to both dicotyledonous and monocotyledonous plants, including *Agrobacterium*-recalcitrant cereals and legumes; stable and transient expression; simultaneous delivery of multiple genetic elements; organelle transformation, including chloroplast transformation	multiple-copy insertion; rearranged transgenes; integration at multiple genomic locations; off-target deletions; possible tissue damage or compromised DNA integrity during delivery
protoplast transformation	simple and reproducible workflow; suitability for transient expression assays, gene function studies, protein localization and preliminary CRISPR/Cas9 editing screens	restriction mainly to protoplasts or a small number of cell types; species-, organ- and tissue-dependent isolation conditions; difficult and time-consuming regeneration of healthy plants from protoplasts
microinjection	precise delivery into selected cells or protoplasts; direct targeting of cytoplasm or nucleus; independence of the type of plant	labor-intensive and technically demanding procedure; low throughput; requirement for costly specialized equipment, trained personnel and efficient recovery or regeneration systems
nanotechnology-based delivery	non-invasive and diffusive delivery properties; potential alternative for *Agrobacterium*-resistant species; targeted delivery; low toxicity; cargo protection; compatibility with transient and stable genetic modification	emerging technological status; recent development of nanotechnology-based plant gene delivery; insufficient exploration of efficient nanoparticle delivery into plants

**Table 2:** Herbicide resistance genes.

GM Plant	Gene	Source	Desirable Trait	Ref.
maize, soybean, cotton, rice, sweet potato, *Arabidopsis*	*4-hydroxyphenylpyruvate dioxygenase* (*HPPD*)	rice, maize, sweet potato, cotton, *Pseudomonas fluorescens*, *Avena sativa*	resistance to HPPD-inhibiting herbicides, increased resistance to abiotic stresses	[116,117,118,119,120,121,122]
*Eleusine coracana*, *Glycine max*, *Linum usitatissimum*, *Nicotiana plumbaginifolia*, *Nicotiana sylvestris*	*alpha*- and *beta-tubulin*	*Eleusine indica*, *Setaria viridis*, *Lolium rigidum*	resistance to herbicides belonging to the group of the microtubule inhibitors	[123,124]
wheat, *Arabidopsis*	*acetolactate synthase* (*ALS*)	*Schoenoplectiella juncoides*, *Triticum aestivum*, *Beckmannia syzigachne*, *Bromus japonicus*, *Echinochloa phyllopogon*, *Schoenoplectiella juncoides*	resistance to ALS inhibitors	[125,126,127,128,129]
soybean, rice, canola	*5-enolpyruvylshikimate-3-phosphate synthase* (*EPSPS*)	rice, *Eleusine indica* (L.) Gaertn., *Conyza canadensis*, *Amaranthus palmeri*, *Amaranthus tuberculatus*, *Amaranthus hybridus*, *Chloris truncata*, *Lolium perenne* ssp. *multiflorum*, *Agrobacterium* sp. *strain CP4*	resistance to glyphosate	[130,131,132,133,134,135]
maize, soybean, canola, cotton	*phosphinothricin acetyltransferase* (*pat*) *bialaphos resistance* (*bar*)	*pat* gene from *Streptomyces viridiochromogenes* *bar* gene from *Streptomyces hygroscopicus*	resistance to glufosinate	[136,137]
canola, alfalfa, cotton, maize, soybean	*glyphosate N-acyltransferase* (*GAT*)	soil microorganisms from extremely glyphosate-polluted soil	resistance to glyphosate	[138,139,140]
cotton, soybean, maize, *Arabidopsis*	2,4-D degrading enzymes (TfdA, RdpA, SdpA)	TfdA from *Ralstonia eutrophus*, RdpA from *Sphingobium herbicidivorans*, SdpA from *Delftia acidovorans*	resistance to 2,4-dichlorophenoxy acetic acid (2,4-D)	[141,142,143]
rice, *Agrostis stolonifera* L.	*acetyl-CoA carboxylase* (*ACCase*)	*Alopecurus myosuroides*, *Echinochloa crus-galli* (L.) P. Beauv., *Lolium multiflorum* Lam., rice	resistance to ACCase-inhibiting herbicides	[144,145,146,147,148,149]
rice, tobacco, *Arabidopsis*	*psbA*	*Raphanus raphanistrum*, *Lolium perenne* L. ssp. multiflorum (Lam.) Husnot, maize, *Arabidopsis*	resistance to PSII-inhibiting herbicides, improved drought tolerance, enhanced sulfur dioxide tolerance, enhanced plant heat tolerance	[150,151,152,153]
rice, *Arabidopsis*	F-box proteins	rice, *Arabidopsis*	resistance to synthetic auxin herbicides (picloram, dicamba)	[154,155]
maize, soybean, cotton, canola	*dicamba monooxygenase* (*dmo*)	*Pseudomonas maltophilia* strain DI-6	resistance to the herbicide dicamba	[156,157]

**Table 3:** Genes for enhancing biomass.

GM Plant	Gene	Source	Desirable Trait	Ref.
poplar, *Arabidopsis*	*Booster*	poplar	improved photosynthetic efficiency, increase in biomass and seed yield	[165]
rice, *Arabidopsis*, potato, tobacco, tomato	*malate synthase* (*MS*) and *glycolate dehydrogenase* (*GDH*)	*MS* gene from pumpkin and the *GDH* gene from the alga *Chlamydomonas*	reduced photorespiration, increased photosynthetic efficiency, enhanced biomass and yield	[166,167,168,169]
tobacco, *Melia azedarach*, poplar, potato, maize, rice, tomato, *Hibiscus cannabinus* L., *Panicum virgatum* L.	*Gibberellic Acid 20 oxidase* (*GA20ox*)	*Arabidopsis*, *Pinus densiflora*, maize, rice	increased level of active gibberellins in plant tissues, leading to faster growth and greater biomass accumulation	[170,171,172,173]
wheat, tomato, tobacco	*sedoheptulose-1*,*7-bisphosphatase* (*SBPase*)	*Brassica napus*, *Brachypodium distachyon*	enhancing the central metabolic process of carbon fixation, increased photosynthetic rates, increased plant biomass and seed yield, increase in starch accumulation, improved resistance to chilling stress	[174,175]
*Arabidopsis*, tobacco, rice, *Brassica napus*	*DWARF4* (*DWF4*)	*Arabidopsis*, *Echinacea purpurea*	increased seed yield, higher root biomass and root length, tolerance to dehydration and heat stress, resistance to fungal pathogens	[176,177]
rice, canola, wheat, barley	*alanine aminotransferase (AlaAT)*	*Hordeum vulgare*, barley	increased above-ground biomass and seed yield, higher root biomass production	[178,179,180]
*Arabidopsis*, rice, cotton, maize, alfalfa, wheat, barley, creeping bentgrass	*vacuolar pyrophosphatase1* (*VP1*)	*Arabidopsis*, *Thellungiella halophila*	enhanced biomass in shoot and root systems, improved phloem loading and transport, resistance to salt stress, improved drought resistance	[181,182,183,184,185]

**Table 4:** Genes improving resistance to pests and diseases.

GM Plant	Gene	Source	Desirable Trait	Ref.
maize, tobacco, potato	Chitinase genes	*Spodoptera littoralis*, the *Autographa californica* nuclear polyhedrosis virus, *Penicillium ochrochloron* Q-3-1, *Streptomyces griseus* strain HUT6037	resistance against insect pests and fungal diseases	[195,196,197,198]
barley, rice	Antimicrobial peptides (AMPs)	*Drosophila melanogaster*, *Hyalophora* *cecropia*	plant protection from bacteria, fungi and viruses	[199,200,201]
tomato, alfalfa	human lactoferrin (hLf)	human milk	enhanced resistance to the phytopathogens	[202,203,204,205]
maize, cotton, soybean	*Cry* genes	*Bacillus thuringiensis*	resistance to the bollworm, resistance to the western corn rootworm	[206,207,208]
tobacco, potato	*inhibitor of serine proteases* (*ISP*)	buckwheat	resistance to bacteria, insects and fungal pathogens	[209,210]
rice	*Pi21*	rice	partial resistance to *Magnaporthe oryzae*	[211,212,213]
tobacco	*thaumatin*	*Thaumatococcus daniellii*	enhanced tolerance to fungal pathogens and abiotic stresses	[214]
rice, *Camellia sinensis* L. (O.) Kuntze	*Osmotin*	rice, tobacco	increased resistance to *Schizotetranychus oryzae*, improved tolerance to drought stress	[215,216,217]

**Table 5:** Genes enhancing consumer properties.

GM Plant	Gene	Source	Desirable Trait	Ref.
Golden Rice, Golden Rice 2	*phytoene synthase* (*psy*) in combination with *carotene desaturase* (*crtI*)	*psy* from *Narcissus pseudonarcissus* (Golden Rice), *psy* from maize (Golden Rice 2), *crtI* from *Erwinia uredovora*	biosynthesis of provitamin A in the rice endosperm	[224,225]
rice, barley, potato	*granule-bound starch synthase* (*GBSS*) genes	*Fagopyrum tataricum* (L.) Gaertn., barley, rice	altered starch content and structure	[226,227,228]
poplar, alfalfa	*Cinnamoyl-CoA Reductase* (*CCR*) genes	poplar, alfalfa	reduced lignin content	[229,230]

**Table 6:** Genes providing abiotic stress resistance.

GM Plant	Gene	Source	Desirable Trait	Ref.
rice, *Osteospermum ecklonis*, *Malus pumila* Mill., *Arabidopsis*	*myb4*	rice	resistance to cold, drought, and even certain diseases, changes in metabolite accumulation	[238,239,240]
*Arabidopsis*, tomato, wheat	*Zat12*	*Arabidopsis*, *Brassica carinata*	resistance to drought and low temperatures	[241,242,243]
tomato, *Arabidopsis*	*cold responsive-element binding factor 3* (*CBF3*)	*Arabidopsis*, *Punica granatum*, *Cuphea hookeriana*	cold resistance	[244,245,246]
wheat, tobacco, *Arabidopsis*	*Dehydration-Responsive Element-Binding 3* (*DREB3)*	soybean	increased drought tolerance	[247,248]

## Data Availability

All inquiries can be directed to the corresponding author.

## Abbreviations

The following abbreviations are used in this manuscript:

GM	genetically modified
T-DNA	transfer DNA
Ti	tumour-inducing
(+)ssRNA	positive-strand
(−)ssRNA	negative-strand
VIGS	virus-induced gene silencing
VIGE	virus-induced genome editing
TSR	target-site resistance
NTSR	non-target-site resistance
AMPs	antimicrobial peptides
Bt	*Bacillus thuringiensis*
ISP	inhibitor of serine protease

## References

[1] Atkins P.A., Voytas D.F. (2020). Overcoming Bottlenecks in Plant Gene Editing. Curr. Opin. Plant Biol..

[2] Jhansi Rani S., Usha R. (2013). Transgenic Plants: Types, Benefits, Public Concerns and Future. J. Pharm. Res..

[3] Barton K.A., Binns A.N., Matzke A.J.M., Chilton M.-D. (1983). Regeneration of Intact Tobacco Plants Containing Full Length Copies of Genetically Engineered T-DNA, and Transmission of T-DNA to R1 Progeny. Cell.

[4] Khan S., Ullah M.W., Siddique R., Nabi G., Manan S., Yousaf M., Hou H. (2016). Role of Recombinant DNA Technology to Improve Life. Int. J. Genom..

[5] Hamdan M.F., Tan B.C. (2025). Genetic Modification Techniques in Plant Breeding: A Comparative Review of CRISPR/Cas and GM Technologies. Hortic. Plant J..

[6] Lacroix B., Citovsky V. (2020). Biolistic Approach for Transient Gene Expression Studies in Plants.

[7] Sangwan R.S., Ochatt S., Nava-Saucedo J.E., Sangwan-Norreel B., Shu Q.Y., Forster B.P., Nakagawa H. (2012). T-DNA Insertion Mutagenesis. Plant Mutation Breeding and Biotechnology.

[8] Lee L.-Y., Gelvin S.B. (2008). T-DNA Binary Vectors and Systems. Plant Physiol..

[9] Leng C., Sun B., Liu Z., Zhang L., Wei X., Zhou Y., Meng Y., Lai Y., Dai Y., Zhu Z. (2020). An Optimized Double T-DNA Binary Vector System for Improved Production of Marker-Free Transgenic Tobacco Plants. Biotechnol. Lett..

[10] Pasin F., Bedoya L.C., Bernabé-Orts J.M., Gallo A., Simón-Mateo C., Orzaez D., García J.A. (2017). Multiple T-DNA Delivery to Plants Using Novel Mini Binary Vectors with Compatible Replication Origins. ACS Synth. Biol..

[11] Goralogia G.S., Willig C., Strauss S.H. (2025). Engineering *Agrobacterium* for Improved Plant Transformation. Plant J..

[12] Srinivasan R., Gothandam K.M. (2016). Synergistic Action of D-Glucose and Acetosyringone on *Agrobacterium* Strains for Efficient *Dunaliella* Transformation. PLoS ONE.

[13] Wang S., Chen H., Wang Y., Pan C., Tang X., Zhang H., Chen W., Chen Y.Q. (2020). Effects of *Agrobacterium tumefaciens* Strain Types on the *Agrobacterium*-Mediated Transformation Efficiency of Filamentous Fungus *Mortierella alpina*. Lett. Appl. Microbiol..

[14] Krishnamohan A., Balaji V., Veluthambi K. (2001). Efficient Vir Gene Induction in *Agrobacterium tumefaciens* Requires virA, virG, and Vir Box from the Same Ti Plasmid. J. Bacteriol..

[15] Fan X., Sun H. (2024). Exploring *Agrobacterium*-Mediated Genetic Transformation Methods and Its Applications in *Lilium*. Plant Methods.

[16] Movahedi A., Zhang J., Amirian R., Zhuge Q. (2014). An Efficient *Agrobacterium*-Mediated Transformation System for Poplar. Int. J. Mol. Sci..

[17] Stachel S.E., Messens E., Van Montagu M., Zambryski P. (1985). Identification of the Signal Molecules Produced by Wounded Plant Cells That Activate T-DNA Transfer in *Agrobacterium tumefaciens*. Nature.

[18] Utami E.S.W., Hariyanto S., Manuhara Y.S.W. (2018). *Agrobacterium tumefaciens*-Mediated Transformation of *Dendrobium Lasianthera* J.J.Sm: An Important Medicinal Orchid. J. Genet. Eng. Biotechnol..

[19] Hwang H.-H., Yu M., Lai E.-M. (2017). *Agrobacterium*-Mediated Plant Transformation: Biology and Applications. Arab. Book.

[20] Altpeter F., Springer N.M., Bartley L.E., Blechl A.E., Brutnell T.P., Citovsky V., Conrad L.J., Gelvin S.B., Jackson D.P., Kausch A.P. (2016). Advancing Crop Transformation in the Era of Genome Editing. Plant Cell.

[21] Long Y., Yang Y., Pan G., Shen Y. (2022). New Insights into Tissue Culture Plant-Regeneration Mechanisms. Front. Plant Sci..

[22] Ikeuchi M., Favero D.S., Sakamoto Y., Iwase A., Coleman D., Rymen B., Sugimoto K. (2019). Molecular Mechanisms of Plant Regeneration. Annu. Rev. Plant Biol..

[23] Fehér A. (2019). Callus, Dedifferentiation, Totipotency, Somatic Embryogenesis: What These Terms Mean in the Era of Molecular Plant Biology?. Front. Plant Sci..

[24] Wang P., Si H., Li C., Xu Z., Guo H., Jin S., Cheng H. (2025). Plant Genetic Transformation: Achievements, Current Status and Future Prospects. Plant Biotechnol. J..

[25] Wang Y.K., Wang Y.P., Zhou L.Z. (2026). Molecular Regulators of Regeneration and Strategies for Overcoming Genotype-Dependent Recalcitrance in Wheat (*Triticum aestivum*). Commun. Biol..

[26] Mountourakis F., Fragkostefanakis S., Moschou P.N. (2026). Protocol for Generating and Selecting Transgenic Tomato Lines through *Agrobacterium tumefaciens*-Mediated Transformation. STAR Protoc..

[27] Szarzanowicz M.J., Waldburger L.M., Busche M., Geiselman G.M., Kirkpatrick L.D., Kehl A.J., Tahmin C., Kuo R.C., McCauley J., Pannu H. (2025). Binary Vector Copy Number Engineering Improves *Agrobacterium*-Mediated Transformation. Nat. Biotechnol..

[28] Fuad F.A.A., Ismail I., Sidik N.M., Zain C.R.C.M., Abdullah R. (2008). Super Binary Vector System Enhanced Transformation Frequency and Expression Level of Polyhydroxyvalerate Gene in Oil Palm Immature Embryo. Asian J. Plant Sci..

[29] Komori T., Imayama T., Kato N., Ishida Y., Ueki J., Komari T. (2007). Current Status of Binary Vectors and Superbinary Vectors. Plant Physiol..

[30] Bélanger J.G., Copley T.R., Hoyos-Villegas V., Charron J.-B., O’Donoughue L. (2024). A Comprehensive Review of in Planta Stable Transformation Strategies. Plant Methods.

[31] Kumar S., Vishwakarma H., Ghosh G., Singh J., Padaria J.C. (2024). In Planta Transformation in Wheat: An Improved Protocol to Develop Wheat Transformants. Mol. Biol. Rep..

[32] Mei G., Chen A., Wang Y., Li S., Wu M., Hu Y., Liu X., Hou X. (2024). A Simple and Efficient in Planta Transformation Method Based on the Active Regeneration Capacity of Plants. Plant Commun..

[33] Tague B.W., Mantis J. (2006). In Planta Agrobacterium-Mediated Transformation by Vacuum Infiltration.

[34] Zhong H., Li C., Yu W., Zhou H., Lieber T., Su X., Wang W., Bumann E., Lunny Castro R.M., Jiang Y. (2024). A Fast and Genotype-Independent in Planta *Agrobacterium*-Mediated Transformation Method for Soybean. Plant Commun..

[35] Hu D., Bent A.F., Hou X., Li Y. (2019). *Agrobacterium*-Mediated Vacuum Infiltration and Floral Dip Transformation of Rapid-Cycling *Brassica Rapa*. BMC Plant Biol..

[36] Bastaki N.K., Cullis C.A. (2014). Floral-Dip Transformation of Flax (*Linum usitatissimum*) to Generate Transgenic Progenies with a High Transformation Rate. J. Vis. Exp..

[37] Clough S.J., Bent A.F. (1998). Floral Dip: A Simplified Method for *Agrobacterium*-mediated Transformation of *Arabidopsis thaliana*. Plant J..

[38] Ali I., Sher H., Ali A., Hussain S., Ullah Z. (2022). Simplified Floral Dip Transformation Method of *Arabidopsis thaliana*. J. Microbiol. Methods.

[39] Wang G., Pantha P., Tran K.-N., Oh D.-H., Dassanayake M. (2019). Plant Growth and *Agrobacterium*-Mediated Floral-Dip Transformation of the Extremophyte *Schrenkiella parvula*. J. Vis. Exp..

[40] Yaroshko O., Vasylenko M., Gajdošová A., Morgun B., Khrystan O., Velykozhon L., Kuchuk M. (2019). “Floral-Dip” Transformation of *Amaranthus caudatus* L. and Hybrids *A. caudatus* × *A. paniculatus* L.. Biologija.

[41] Purwantoro A., Irsyadi M.B., Sawitri W.D., Fatumi N.C., Fajrina S.N. (2023). Efficient Floral Dip Transformation Method Using *Agrobacterium tumefaciens* on *Cosmos sulphureus* Cav. Saudi J. Biol. Sci..

[42] Mu G., Chang N., Xiang K., Sheng Y., Zhang Z., Pan G. (2012). Genetic Transformation of Maize Female Inflorescence Following Floral Dip Method Mediated by *Agrobacterium*. Biotechnology.

[43] Ratanasut K., Rod-In W., Sujipuli K. (2017). In Planta *Agrobacterium*-Mediated Transformation of Rice. Rice Sci..

[44] Bechtold N., Pelletier G. (1998). In Planta Agrobacterium-Mediated Transformation of Adult Arabidopsis Thaliana Plants by Vacuum Infiltration.

[45] Joh L.D., Wroblewski T., Ewing N.N., VanderGheynst J.S. (2005). High-level Transient Expression of Recombinant Protein in Lettuce. Biotech. Bioeng..

[46] Wang W.C., Menon G., Hansen G. (2003). Development of a Novel *Agrobacterium*-Mediated Transformation Method to Recover Transgenic *Brassica Napus* Plants. Plant Cell Rep..

[47] Subramanyam K., Subramanyam K., Sailaja K.V., Srinivasulu M., Lakshmidevi K. (2011). Highly Efficient *Agrobacterium*-Mediated Transformation of Banana Cv. Rasthali (AAB) via Sonication and Vacuum Infiltration. Plant Cell Rep..

[48] Arun M., Subramanyam K., Mariashibu T.S., Theboral J., Shivanandhan G., Manickavasagam M., Ganapathi A. (2015). Application of Sonication in Combination with Vacuum Infiltration Enhances the *Agrobacterium*-Mediated Genetic Transformation in Indian Soybean Cultivars. Appl. Biochem. Biotechnol..

[49] Amal T.C., Karthika P., Dhandapani G., Selvakumar S., Vasanth K. (2020). A Simple and Efficient *Agrobacterium*-Mediated in Planta Transformation Protocol for Horse Gram (*Macrotyloma Uniflorum* Lam. Verdc.). J. Genet. Eng. Biotechnol..

[50] Bett B., Gollasch S., Moore A., Harding R., Higgins T.J.V. (2019). An Improved Transformation System for Cowpea (*Vigna Unguiculata* L. Walp) via Sonication and a Kanamycin-Geneticin Selection Regime. Front. Plant Sci..

[51] Sivanandhan G., Kapil Dev G., Theboral J., Selvaraj N., Ganapathi A., Manickavasagam M. (2015). Sonication, Vacuum Infiltration and Thiol Compounds Enhance the *Agrobacterium*-Mediated Transformation Frequency of *Withania somnifera* (L.) Dunal. PLoS ONE.

[52] Hao Y., Yuan Z., Li Y., Zuo D., Cheng H., Wang Q., Zhang Y., Lv L., Liu J., Song G. (2025). An All-in-One Plant Virus-Based Vector Toolkit for Streamlined Gene Silencing, Overexpression, and Genome Editing. Plant Commun..

[53] Mahmood M.A., Naqvi R.Z., Rahman S.U., Amin I., Mansoor S. (2023). Plant Virus-Derived Vectors for Plant Genome Engineering. Viruses.

[54] Shen Y., Ye T., Li Z., Kimutai T.H., Song H., Dong X., Wan J. (2024). Exploiting Viral Vectors to Deliver Genome Editing Reagents in Plants. aBIOTECH.

[55] Abrahamian P., Hammond R.W., Hammond J. (2020). Plant Virus-Derived Vectors: Applications in Agricultural and Medical Biotechnology. Annu. Rev. Virol..

[56] Cheuk A., Houde M. (2018). A New Barley Stripe Mosaic Virus Allows Large Protein Overexpression for Rapid Function Analysis. Plant Physiol..

[57] Kaya H., Ishibashi K., Toki S. (2017). A Split Staphylococcus Aureus Cas9 as a Compact Genome-Editing Tool in Plants. Plant Cell Physiol..

[58] Gao Q., Xu W., Yan T., Fang X., Cao Q., Zhang Z., Ding Z., Wang Y., Wang X. (2019). Rescue of a Plant Cytorhabdovirus as Versatile Expression Platforms for Planthopper and Cereal Genomic Studies. New Phytol..

[59] Liu Q., Zhao C., Sun K., Deng Y., Li Z. (2023). Engineered Biocontainable RNA Virus Vectors for Non-Transgenic Genome Editing across Crop Species and Genotypes. Mol. Plant.

[60] Ma X., Zhang X., Liu H., Li Z. (2020). Highly Efficient DNA-Free Plant Genome Editing Using Virally Delivered CRISPR-Cas9. Nat. Plants.

[61] Jackson A.O., Li Z. (2016). Developments in Plant Negative-Strand RNA Virus Reverse Genetics. Annu. Rev. Phytopathol..

[62] Bradamante G., Mittelsten Scheid O., Incarbone M. (2021). Under Siege: Virus Control in Plant Meristems and Progeny. Plant Cell.

[63] Fiallo-Olivé E., Lett J.-M., Martin D.P., Roumagnac P., Varsani A., Zerbini F.M., Navas-Castillo J. (2021). ICTV Virus Taxonomy Profile: *Geminiviridae* 2021. J. Gen. Virol..

[64] Zhang Y., Deng S. (2026). Geminivirus Vectors: From Gene Silencing to Synthetic Biology. Biotechnol. Adv..

[65] Thomas J.E., Gronenborn B., Harding R.M., Mandal B., Grigoras I., Randles J.W., Sano Y., Timchenko T., Vetten H.J., Yeh H.-H. (2021). ICTV Virus Taxonomy Profile: *Nanoviridae*. J. Gen. Virol..

[66] Dasgupta I., Khan Z.A., Bhat A.I., Viswanathan R., Sastry K.S., Rao G.P., Sastry K.S., Debat H., Maruthi M.N., Dasgupta I., Rao G.P., Bhat A.I., Venkataravanappa V., Reddy M.K., Viswanathan R. (2026). Taxonomy of Family: *Caulimoviridae*. Taxonomy and Classification of Plant Viruses and Viroids.

[67] Teycheney P.-Y., Geering A.D.W., Dasgupta I., Hull R., Kreuze J.F., Lockhart B., Muller E., Olszewski N., Pappu H., Pooggin M.M. (2020). ICTV Virus Taxonomy Profile: *Caulimoviridae*. J. Gen. Virol..

[68] Baltes N.J., Gil-Humanes J., Cermak T., Atkins P.A., Voytas D.F. (2014). DNA Replicons for Plant Genome Engineering. Plant Cell.

[69] Huang Z., Chen Q., Hjelm B., Arntzen C., Mason H. (2009). A DNA Replicon System for Rapid High-Level Production of Virus-like Particles in Plants. Biotechnol. Bioeng..

[70] Mikhaylova E. (2025). Virus-Induced Genome Editing (VIGE): One Step Away from an Agricultural Revolution. Int. J. Mol. Sci..

[71] Gupta D., Ranjan R. (2017). In Silico Comparative Analysis of Promoters Derived from Plant Pararetroviruses. VirusDisease.

[72] Hohn T. (2013). Plant Pararetroviruses: Interactions of Cauliflower Mosaic Virus with Plants and Insects. Curr. Opin. Virol..

[73] Willemsen A., Zwart M.P. (2019). On the Stability of Sequences Inserted into Viral Genomes. Virus Evol..

[74] Gronenborn B., Gardner R.C., Schaefer S., Shepherd R.J. (1992). Propagation of Foreign DNA in Plants Using Cauliflower Mosaic Virus as Vector. 1981. Biotechnology.

[75] European and Mediterranean Plant Protection Organization 2022 (2022). PM 7/153 (1) Mechanical Inoculation of Test Plants. EPPO Bull..

[76] Ellis M.D., Hoak J.M., Ellis B.W., Brown J.A., Sit T.L., Wilkinson C.A., Reed T.D., Welbaum G.E. (2020). Quantitative Real-Time PCR Analysis of Individual Flue-Cured Tobacco Seeds and Seedlings Reveals Seed Transmission of Tobacco Mosaic Virus. Phytopathology.

[77] Hull R. (2009). Mechanical Inoculation of Plant Viruses. Curr. Protoc. Microbiol..

[78] Monroy-Borrego A.G., Steinmetz N.F. (2022). Three Methods for Inoculation of Viral Vectors into Plants. Front. Plant Sci..

[79] Torti S., Schlesier R., Thümmler A., Bartels D., Römer P., Koch B., Werner S., Panwar V., Kanyuka K., Wirén N.V. (2021). Transient Reprogramming of Crop Plants for Agronomic Performance. Nat. Plants.

[80] Peyret H., Lomonossoff G.P. (2015). When Plant Virology Met *Agrobacterium*: The Rise of the Deconstructed Clones. Plant Biotechnol. J..

[81] Gao C., Nielsen K.K., Sudowe S., Reske-Kunz A.B. (2013). Comparison Between *Agrobacterium*-Mediated and Direct Gene Transfer Using the Gene Gun. Biolistic DNA Delivery.

[82] Sujatha M., Visarada K.B.R.S., Sudowe S., Reske-Kunz A.B. (2013). Transformation of Nuclear DNA in Meristematic and Embryogenic Tissues. Biolistic DNA Delivery.

[83] Thorpe C., Luo W., Ji Q., Eggenberger A.L., Chicowski A.S., Xu W., Sandhu R., Lee K., Whitham S.A., Qi Y. (2025). Enhancing Biolistic Plant Transformation and Genome Editing with a Flow Guiding Barrel. Nat. Commun..

[84] Miller K., Eggenberger A.L., Lee K., Liu F., Kang M., Drent M., Ruba A., Kirscht T., Wang K., Jiang S. (2021). An Improved Biolistic Delivery and Analysis Method for Evaluation of DNA and CRISPR-Cas Delivery Efficacy in Plant Tissue. Sci. Rep..

[85] Lai K.-S., Abdullah P., Yusoff K., Mahmood M. (2011). An Efficient Protocol for Particle Bombardment-Mediated Transformation of *Centella Asiatica* Callus. Acta Physiol. Plant.

[86] Faraco M., Di Sansebastiano G.P., Spelt K., Koes R.E., Quattrocchio F.M. (2011). One Protoplast Is Not the Other!. Plant Physiol..

[87] Davey M.R., Anthony P., Power J.B., Lowe K.C. (2005). Plant Protoplasts: Status and Biotechnological Perspectives. Biotechnol. Adv..

[88] Batool S., Li Z., Zhang D., Shi P., Htwe Y.M., Nie H., Ma M., Su H., Fang X., Ahmed M.A.A. (2025). PEG-Mediated Transformation and CRISPR/Cas9 Gene Editing of CnPDS in Coconut Protoplast. Ind. Crops Prod..

[89] Subburaj S., Agapito-Tenfen S.Z. (2023). Establishment of Targeted Mutagenesis in Soybean Protoplasts Using CRISPR/Cas9 RNP Delivery via Electro−transfection. Front. Plant Sci..

[90] Wang H., Wang W., Zhan J., Huang W., Xu H. (2015). An Efficient PEG-Mediated Transient Gene Expression System in Grape Protoplasts and Its Application in Subcellular Localization Studies of Flavonoids Biosynthesis Enzymes. Sci. Hortic..

[91] Hsu Y.-Y., Chen S.J., Bernal-Chanchavac J., Sharma B., Moghimianavval H., Stephanopoulos N., Liu A.P. (2023). Calcium-Triggered DNA-Mediated Membrane Fusion in Synthetic Cells. Chem. Commun..

[92] Ozyigit I.I. (2020). Gene Transfer to Plants by Electroporation: Methods and Applications. Mol. Biol. Rep..

[93] Duarte P., Ribeiro D., Carqueijeiro I., Bettencourt S., Sottomayor M., Fett-Neto A.G. (2016). Protoplast Transformation as a Plant-Transferable Transient Expression System. Biotechnology of Plant Secondary Metabolism.

[94] Neuhaus G., Spangenberg G. (1990). Plant Transformation by Microinjection Techniques. Physiol. Plant..

[95] Schnorf M., Neuhaus-Url G., Galli A., Iida S., Potrykus I., Neuhaus G. (1991). An Improved Approach for Transformation of Plant Cells by Microinjection: Molecular and Genetic Analysis. Transgenic Res..

[96] Su W., Xu M., Radani Y., Yang L. (2023). Technological Development and Application of Plant Genetic Transformation. Int. J. Mol. Sci..

[97] Shivashakarappa K., Marriboina S., Dumenyo K., Taheri A., Yadegari Z. (2025). Nanoparticle-Mediated Gene Delivery Techniques in Plant Systems. Front. Nanotechnol..

[98] Yan Y., Zhu X., Yu Y., Li C., Zhang Z., Wang F. (2022). Nanotechnology Strategies for Plant Genetic Engineering. Adv. Mater..

[99] Lv Z., Jiang R., Chen J., Chen W. (2020). Nanoparticle-mediated Gene Transformation Strategies for Plant Genetic Engineering. Plant J..

[100] Chen J.-T. (2026). Nano-Delivering for Plant Genetic Engineering.

[101] Sisea C.-R., Seifi M. (2024). Characteristics of Various Types of Plant Breeding. Genetics.

[102] Kalra B., Priyanka, Sharma V., Prudhvinadh G., Sonia, Rajan R., Ahmad F., Pandey K. (2025). Implication of Molecular Techniques for Sustainable Crop Improvement. Innovations in Climate Resilient Agriculture.

[103] Gilbertson L., Puchta H., Slotkin R.K. (2025). The Future of Genome Editing in Plants. Nat. Plants.

[104] Zhang R., Zheng Z., Li G., Zheng X., Su L., Yuan X., Li T., Tan J., Zeng D., Zhang S. (2026). Plant Base Editing: A Decade of Progress and Future Applications. aBIOTECH.

[105] Binenbaum J., Adamkova V., Fryer H., Xu L., Gorringe N., Włodzimierz P., Burns R., Papikian A., Jacobsen S.E., Henderson I.R. (2025). CRISPR Targeting of H3K4me3 Activates Gene Expression and Unlocks Centromere-Proximal Crossover Recombination in *Arabidopsis*. Nat. Commun..

[106] Délye C., Jasieniuk M., Le Corre V. (2013). Deciphering the Evolution of Herbicide Resistance in Weeds. Trends Genet..

[107] Gaines T.A., Duke S.O., Morran S., Rigon C.A.G., Tranel P.J., Küpper A., Dayan F.E. (2020). Mechanisms of Evolved Herbicide Resistance. J. Biol. Chem..

[108] Palma-Bautista C., Belluccini P., Vázquez-García J.G., Alcántara-de La Cruz R., Barro F., Portugal J., De Prado R. (2023). Target-Site and Non-Target-Site Resistance Mechanisms Confer Multiple Resistance to Glyphosate and 2,4-D in *Carduus acanthoides*. Pestic. Biochem. Physiol..

[109] Kutasy B., Farkas Z., Kolics B., Decsi K., Hegedűs G., Kovács J., Taller J., Tóth Z., Kálmán N., Kazinczi G. (2021). Detection of Target-Site Herbicide Resistance in the Common Ragweed: Nucleotide Polymorphism Genotyping by Targeted Amplicon Sequencing. Diversity.

[110] Nandula V.K., Giacomini D.A., Molin W.T. (2020). Target Site-Based Resistance to ALS Inhibitors, Glyphosate, and PPO Inhibitors in an *Amaranthus palmeri* Accession from Mississippi. Am. J. Plant Sci..

[111] Gaines T.A., Zhang W., Wang D., Bukun B., Chisholm S.T., Shaner D.L., Nissen S.J., Patzoldt W.L., Tranel P.J., Culpepper A.S. (2010). Gene Amplification Confers Glyphosate Resistance in *Amaranthus palmeri*. Proc. Natl. Acad. Sci. USA.

[112] Panozzo S., Mascanzoni E., Scarabel L., Milani A., Dalazen G., Merotto A.J., Tranel P.J., Sattin M. (2021). Target-Site Mutations and Expression of *ALS* Gene Copies Vary According to *Echinochloa* Species. Genes.

[113] Franco-Ortega S., Goldberg-Cavalleri A., Walker A., Brazier-Hicks M., Onkokesung N., Edwards R. (2021). Non-Target Site Herbicide Resistance Is Conferred by Two Distinct Mechanisms in Black-Grass (*Alopecurus myosuroides*). Front. Plant Sci..

[114] Jugulam M., Shyam C. (2019). Non-Target-Site Resistance to Herbicides: Recent Developments. Plants.

[115] Suzukawa A.K., Bobadilla L.K., Mallory-Smith C., Brunharo C.A.C.G. (2021). Non-Target-Site Resistance in *Lolium* Spp. Globally: A Review. Front. Plant Sci..

[116] Wu Y., Xiao N., Cai Y., Yang Q., Yu L., Chen Z., Shi W., Liu J., Pan C., Li Y. (2023). CRISPR-Cas9-Mediated Editing of the OsHPPD 3′ UTR Confers Enhanced Resistance to HPPD-Inhibiting Herbicides in Rice. Plant Commun..

[117] Zhao B., Wang Y., Zhou M., Liu X., Li H., Shi Z., Guo Y., Li T., Yang F., Wang R. (2025). Identification and Application of Herbicide-resistant 4-hydroxyphenylpyruvate Dioxygenase (HPPD) Alleles via Directed Evolution. Plant Biotechnol. J..

[118] Kim S.-E., Bian X., Lee C.-J., Park S.-U., Lim Y.-H., Kim B.H., Park W.S., Ahn M.-J., Ji C.Y., Yu Y. (2021). Overexpression of 4-Hydroxyphenylpyruvate Dioxygenase (IbHPPD) Increases Abiotic Stress Tolerance in Transgenic Sweetpotato Plants. Plant Physiol. Biochem..

[119] Qian H., Shi H. (2024). Herbicide-Resistant 4-Hydroxyphenylpyruvate Dioxygenase Variants Identified via Directed Evolution. J. Exp. Bot..

[120] Dreesen R., Capt A., Oberdoerfer R., Coats I., Pallett K.E. (2018). Characterization and Safety Evaluation of HPPD W336, a Modified 4-Hydroxyphenylpyruvate Dioxygenase Protein, and the Impact of Its Expression on Plant Metabolism in Herbicide-Tolerant MST-FGØ72-2 Soybean. Regul. Toxicol. Pharmacol..

[121] Hawkes T.R., Langford M.P., Viner R., Blain R.E., Callaghan F.M., Mackay E.A., Hogg B.V., Singh S., Dale R.P. (2019). Characterization of 4-Hydroxyphenylpyruvate Dioxygenases, Inhibition by Herbicides and Engineering for Herbicide Tolerance in Crops. Pestic. Biochem. Physiol..

[122] Matringe M., Sailland A., Pelissier B., Rolland A., Zink O. (2005). *p*-Hydroxyphenylpyruvate Dioxygenase Inhibitor-resistant Plants. Pest. Manag. Sci..

[123] Chen J., Yu Q., Patterson E., Sayer C., Powles S. (2021). Dinitroaniline Herbicide Resistance and Mechanisms in Weeds. Front. Plant Sci..

[124] Blume Y.B., Strashnyuk N.M., Smertenko A.P., Solodushko V.G., Sidorov V.A., Gleba Y.Y. (1998). Alteration of β-Tubulin in *Nicotiana Plumbaginifolia* Confers Resistance to Amiprophos-Methyl. Theor. Appl. Genet..

[125] Wang J., Qi J., Ouyang Y., Zhou S., Qin L., Zhang B., Bai L., Pan L. (2024). The Mutation Asp-376-Glu in the ALS Gene Confers Resistance to Mesosulfuron-Methyl in *Beckmannia syzigachne*. Plant Physiol. Biochem..

[126] Xu X., Xu J., Zhao B., Guo B., Wei S., Li B., Qi Z., Chen S., Wang G., Liu X. (2026). Target-Site Mutations and Non-Target-Site Detoxification Confer ALS-Inhibitor Resistance in *Bromus Japonicus* Populations in China. Pest. Manag. Sci..

[127] Zhang L., Du Y., Deng Y., Bai T., Wang J., Wang W., Ji M. (2024). Mutations in Target Gene Confers Resistance to Acetolactate Synthase Inhibitors in *Echinochloa phyllopogon*. Plant Physiol. Biochem..

[128] Kumar P., Bishnoi R., Priyadarshini P., Chhuneja P., Singla D. (2025). Understanding the Structural Basis of ALS Mutations Associated with Resistance to Sulfonylurea in Wheat. Sci. Rep..

[129] Ohta K., Kawamata E., Hori T., Sada Y. (2024). Connecting Genes to Whole Plants in Dilution Effect of Target-Site ALS Inhibitor Resistance of *Schoenoplectiella Juncoides* (Roxb.) Lye (*Cyperaceae*). Pestic. Biochem. Physiol..

[130] Achary V.M.M., Sheri V., Manna M., Panditi V., Borphukan B., Ram B., Agarwal A., Fartyal D., Teotia D., Masakapalli S.K. (2020). Overexpression of Improved *EPSPS* Gene Results in Field Level Glyphosate Tolerance and Higher Grain Yield in Rice. Plant Biotechnol. J..

[131] Chen J., Li Z., Cui H., Yu H., Li X. (2023). Gene Amplification of EPSPS with a Mutation in Conserved Region: The Evolved Glyphosate Resistance Mechanism in *Eleusine indica*. Agronomy.

[132] Perotti V.E., Larran A.S., Palmieri V.E., Martinatto A.K., Alvarez C.E., Tuesca D., Permingeat H.R. (2019). A Novel Triple Amino Acid Substitution in the EPSPS Found in a High-level Glyphosate-resistant *Amaranthus hybridus* Population from Argentina. Pest. Manag. Sci..

[133] Islam M.M., Gill B.S., Malone J.M., Preston C., Jugulam M. (2025). Ectopic Recombination: A Novel Mechanism of *EPSPS* Gene Amplification in Glyphosate-resistant *Chloris truncata*. Plant J..

[134] Salas R.A., Dayan F.E., Pan Z., Watson S.B., Dickson J.W., Scott R.C., Burgos N.R. (2012). EPSPS Gene Amplification in Glyphosate-Resistant Italian Ryegrass (*Lolium perenne* ssp. Multiflorum) from Arkansas. Pest. Manag. Sci..

[135] Wu H., Zhang Y., Zhu C., Xiao X., Zhou X., Xu S., Shen W., Huang M. (2012). Presence of CP4-EPSPS Component in Roundup Ready Soybean-Derived Food Products. Int. J. Mol. Sci..

[136] Sivamani E., Nalapalli S., Prairie A., Bradley D., Richbourg L., Strebe T., Liebler T., Wang D., Que Q. (2019). A Study on Optimization of Pat Gene Expression Cassette for Maize Transformation. Mol. Biol. Rep..

[137] Wehrmann A., Vliet A.V., Opsomer C., Botterman J., Schulz A. (1996). The Similarities of Bar and Pat Gene Products Make Them Equally Applicable for Plant Engineers. Nat. Biotechnol..

[138] Liang C., Sun B., Meng Z., Meng Z., Wang Y., Sun G., Zhu T., Lu W., Zhang W., Malik W. (2017). Co-Expression of GR79 EPSPS and GAT Yields Herbicide-Resistant Cotton with Low Glyphosate Residues. Plant Biotechnol. J..

[139] Li S., Li P., Li X., Wen N., Wang Y., Lu W., Lin M., Lang Z. (2023). In Maize, Co-Expression of GAT and GR79-EPSPS Provides High Glyphosate Resistance, along with Low Glyphosate Residues. aBIOTECH.

[140] Yang M., Wen Z., Fazal A., Hua X., Xu X., Yin T., Qi J., Yang R., Lu G., Hong Z. (2020). Impact of a *G2-EPSPS* & *GAT* Dual Transgenic Glyphosate-Resistant Soybean Line on the Soil Microbial Community under Field Conditions Affected by Glyphosate Application. Microbes Environ..

[141] Charles G.W., Constable G.A., Llewellyn D.J., Hickman M.A. (2007). Tolerance of Cotton Expressing a 2,4-D Detoxification Gene to 2,4-D Applied in the Field. Aust. J. Agric. Res..

[142] Wright T.R., Shan G., Walsh T.A., Lira J.M., Cui C., Song P., Zhuang M., Arnold N.L., Lin G., Yau K. (2010). Robust Crop Resistance to Broadleaf and Grass Herbicides Provided by Aryloxyalkanoate Dioxygenase Transgenes. Proc. Natl. Acad. Sci. USA.

[143] Laurent F., Debrauwer L., Rathahao E., Scalla R. (2000). 2,4-Dichlorophenoxyacetic Acid Metabolism in Transgenic Tolerant Cotton (*Gossypium hirsutum*). J. Agric. Food Chem..

[144] Délye C., Michel S., Bérard A., Chauvel B., Brunel D., Guillemin J.-P., Dessaint F., Le Corre V. (2010). Geographical Variation in Resistance to Acetyl-Coenzyme A Carboxylase-Inhibiting Herbicides across the Range of the Arable Weed *Alopecurus Myosuroides* (Black-Grass). New Phytol..

[145] Délye C., Menchari Y., Guillemin J., Matéjicek A., Michel S., Camilleri C., Chauvel B. (2007). Status of Black Grass (*Alopecurus myosuroides*) Resistance to Acetyl-coenzyme A Carboxylase Inhibitors in France. Weed Res..

[146] Hu W., Li Y., Zhao C., Zhang L., Ma Y., Gao Q., Cao H., Liao M. (2025). A Cys-2088-Arg Mutation in *ACCase* Confers Cross-Resistance to ACCase-Inhibiting Herbicides in Barnyardgrass (*Echinochloa Crus-Galli*). Weed Sci..

[147] Heckart D.L., Schwartz B.M., Raymer P.L., Parrott W.A. (2016). Synonymous Mutation Gene Design to Overexpress ACCase in Creeping Bentgrass to Obtain Resistance to ACCase-Inhibiting Herbicides. Transgenic Res..

[148] Rangani G., Langaro A.C., Agrawal S., Salas-Perez R.A., Velásquez J.C., Nelson C.E., Roma-Burgos N. (2024). Resistance to Acetyl Coenzyme A Carboxylase (ACCase) Inhibitor in *Lolium Multiflorum*: Effect of Multiple Target-Site Mutations. Agronomy.

[149] Wang H., He Y., Wang Y., Li Z., Hao J., Song Y., Wang M., Zhu J. (2022). Base Editing-mediated Targeted Evolution of ACCase for Herbicide-resistant Rice Mutants. JIPB.

[150] Lu H., Yu Q., Han H., Owen M.J., Powles S.B. (2019). A Novel psbA Mutation (Phe274-Val) Confers Resistance to PSII Herbicides in Wild Radish (*Raphanus raphanistrum*). Pest. Manag. Sci..

[151] Yin S., Mukaremu N., Zhang M., Chen Z., Wen Y., Dong L., Feng Z. (2026). *psbA* Gene Overexpression Induces Isoproturon Resistance in Italian Ryegrass (*Lolium perenne* ssp. *multiflorum*). Weed Sci..

[152] Huo Y., Wang M., Wei Y., Xia Z. (2015). Overexpression of the Maize psbA Gene Enhances Drought Tolerance Through Regulating Antioxidant System, Photosynthetic Capability, and Stress Defense Gene Expression in Tobacco. Front. Plant Sci..

[153] Su X., Zhou P., Wang R., Luo Z., Xia Z. (2015). Overexpression of the Maize psbA Gene Enhances Sulfur Dioxide Tolerance in Transgenic Tobacco. Plant Cell Tiss. Organ. Cult..

[154] Guo F., Huang Y., Qi P., Lian G., Hu X., Han N., Wang J., Zhu M., Qian Q., Bian H. (2021). Functional Analysis of Auxin Receptor *OsTIR1*/*OsAFB* Family Members in Rice Grain Yield, Tillering, Plant Height, Root System, Germination, and Auxinic Herbicide Resistance. New Phytol..

[155] Wei H., Li D., Xie K., Lou S., Dong G., Guo F., Lian G., Pan X., Zeng Z., Han N. (2025). Creation of New Rice Germplasm with Cross-Resistance to Auxin Herbicides Picloram and Dicamba by Genome Editing of OsAFB4. Theor. Appl. Genet..

[156] Cao M., Sato S.J., Behrens M., Jiang W.Z., Clemente T.E., Weeks D.P. (2011). Genetic Engineering of Maize (*Zea mays*) for High-Level Tolerance to Treatment with the Herbicide Dicamba. J. Agric. Food Chem..

[157] Taylor M., Bickel A., Mannion R., Bell E., Harrigan G.G. (2017). Dicamba-Tolerant Soybeans (*Glycine max* L.) MON 87708 and MON 87708 × MON 89788 Are Compositionally Equivalent to Conventional Soybean. J. Agric. Food Chem..

[158] Zhigailov A.V., Stanbekova G.E., Beisenov D.K., Nizkorodova A.S., Polimbetova N.S., Iskakov B.K. (2020). Constructing the Constitutively Active Ribosomal Protein S6 Kinase 2 from *Arabidopsis Thaliana* (AtRPS6K2) and Testing Its Activity in Vitro. Vavilovskii Zhurnal Genet. Sel..

[159] Yu Q., Liu S., Yu L., Xiao Y., Zhang S., Wang X., Xu Y., Yu H., Li Y., Yang J. (2021). RNA Demethylation Increases the Yield and Biomass of Rice and Potato Plants in Field Trials. Nat. Biotechnol..

[160] Jin Y.-L., Tang R.-J., Wang H.-H., Jiang C.-M., Bao Y., Yang Y., Liang M.-X., Sun Z.-C., Kong F.-J., Li B. (2017). Overexpression of *Populus Trichocarpa* CYP85A3 Promotes Growth and Biomass Production in Transgenic Trees. Plant Biotechnol. J..

[161] Xu H., Sun H., Dong J., Ma C., Li J., Li Z., Wang Y., Ji J., Hu X., Wu M. (2022). The Brassinosteroid Biosynthesis Gene TaD11-2A Controls Grain Size and Its Elite Haplotype Improves Wheat Grain Yields. Theor. Appl. Genet..

[162] Fan X., Xie D., Chen J., Lu H., Xu Y., Ma C., Xu G. (2014). Over-Expression of *OsPTR6* in Rice Increased Plant Growth at Different Nitrogen Supplies but Decreased Nitrogen Use Efficiency at High Ammonium Supply. Plant Sci..

[163] Qu B., He X., Wang J., Zhao Y., Teng W., Shao A., Zhao X., Ma W., Wang J., Li B. (2015). A Wheat CCAAT Box-Binding Transcription Factor Increases the Grain Yield of Wheat with Less Fertilizer Input. Plant Physiol..

[164] Yu L.-H., Wu J., Tang H., Yuan Y., Wang S.-M., Wang Y.-P., Zhu Q.-S., Li S.-G., Xiang C.-B. (2016). Overexpression of *Arabidopsis* NLP7 Improves Plant Growth under Both Nitrogen-Limiting and -Sufficient Conditions by Enhancing Nitrogen and Carbon Assimilation. Sci. Rep..

[165] Feyissa B.A., De Becker E.M., Salesse-Smith C.E., Shu M., Zhang J., Yates T.B., Xie M., De K., Gotarkar D., Chen M.S.S. (2025). An Orphan Gene BOOSTER Enhances Photosynthetic Efficiency and Plant Productivity. Dev. Cell.

[166] Chen X., Li G., He H., Xie W., Cui L., Zhang Z., Peng X., Zhu G. (2026). A Synthetic Glycolate Metabolism Bypass in Rice Chloroplasts Increases Photosynthesis and Yield. Crop J..

[167] Nölke G., Houdelet M., Kreuzaler F., Peterhänsel C., Schillberg S. (2014). The Expression of a Recombinant Glycolate Dehydrogenase Polyprotein in Potato (*Solanum tuberosum*) Plastids Strongly Enhances Photosynthesis and Tuber Yield. Plant Biotechnol. J..

[168] Dougherty L., Cooper B., Bunce J., Vinyard B., Stommel J. (2025). Biomass and Yield in *Solanum Lycopersicum* Expressing a Synthetic Photorespiration Pathway. J. Amer. Soc. Hort. Sci..

[169] Smith E.N., Van Aalst M., Tosens T., Niinemets Ü., Stich B., Morosinotto T., Alboresi A., Erb T.J., Gómez-Coronado P.A., Tolleter D. (2023). Improving Photosynthetic Efficiency toward Food Security: Strategies, Advances, and Perspectives. Mol. Plant.

[170] Ly L.K., Bui T.P., Van Thi Le A., Van Nguyen P., Ong P.X., Pham N.B., Zhang Z.J., Do P.T., Chu H.H. (2022). Enhancing Plant Growth and Biomass Production by Overexpression of GA20ox Gene under Control of a Root Preferential Promoter. Transgenic Res..

[171] Fladung M. (2022). Xylem-Specific Overexpression of the GIBBERELLIN ACID 20 OXIDASE Gene (GA20-OXIDASE) from Pine in Hybrid Poplar (*Populus tremula* L. × *P. alba* L.) Revealed Reliable Increase in Growth and Biomass Production Just in a Single-Copy-Line. Gesunde Pflanz..

[172] Voorend W., Nelissen H., Vanholme R., De Vliegher A., Van Breusegem F., Boerjan W., Roldán-Ruiz I., Muylle H., Inzé D. (2016). Overexpression of GA20-OXIDASE1 Impacts Plant Height, Biomass Allocation and Saccharification Efficiency in Maize. Plant Biotechnol. J..

[173] Do P.T., De Tar J.R., Lee H., Folta M.K., Zhang Z.J. (2016). Expression of *Zm GA 20ox* cDNA Alters Plant Morphology and Increases Biomass Production of Switchgrass (*Panicum virgatum* L.). Plant Biotechnol. J..

[174] Driever S.M., Simkin A.J., Alotaibi S., Fisk S.J., Madgwick P.J., Sparks C.A., Jones H.D., Lawson T., Parry M.A.J., Raines C.A. (2017). Increased SBPase Activity Improves Photosynthesis and Grain Yield in Wheat Grown in Greenhouse Conditions. Philos. Trans. R. Soc. Lond. B Biol. Sci..

[175] Ding F., Wang M., Zhang S., Ai X. (2016). Changes in SBPase Activity Influence Photosynthetic Capacity, Growth, and Tolerance to Chilling Stress in Transgenic Tomato Plants. Sci. Rep..

[176] Zhao J., Liu J., He Y., Fu Y. (2025). Development of Semi-Dwarf *Echinacea Purpurea* by Inducing Silencing of the Endogenous Brassinosteroid-Biosynthetic Gene DWF4. BMC Plant Biol..

[177] Sahni S., Prasad B.D., Liu Q., Grbic V., Sharpe A., Singh S.P., Krishna P. (2016). Overexpression of the Brassinosteroid Biosynthetic Gene DWF4 in *Brassica Napus* Simultaneously Increases Seed Yield and Stress Tolerance. Sci. Rep..

[178] Tiong J., Sharma N., Sampath R., MacKenzie N., Watanabe S., Metot C., Lu Z., Skinner W., Lu Y., Kridl J. (2021). Improving Nitrogen Use Efficiency Through Overexpression of Alanine Aminotransferase in Rice, Wheat, and Barley. Front. Plant Sci..

[179] Selvaraj M.G., Valencia M.O., Ogawa S., Lu Y., Wu L., Downs C., Skinner W., Lu Z., Kridl J.C., Ishitani M. (2017). Development and Field Performance of Nitrogen Use Efficient Rice Lines for Africa. Plant Biotechnol. J..

[180] Peña P.A., Quach T., Sato S., Ge Z., Nersesian N., Dweikat I.M., Soundararajan M., Clemente T. (2017). Molecular and Phenotypic Characterization of Transgenic Wheat and Sorghum Events Expressing the Barley Alanine Aminotransferase. Planta.

[181] Khadilkar A.S., Yadav U.P., Salazar C., Shulaev V., Paez-Valencia J., Pizzio G.A., Gaxiola R.A., Ayre B.G. (2016). Constitutive and Companion Cell-Specific Overexpression of AVP1, Encoding a Proton-Pumping Pyrophosphatase, Enhances Biomass Accumulation, Phloem Loading, and Long-Distance Transport. Plant Physiol..

[182] Bao A.-K., Wang S.-M., Wu G.-Q., Xi J.-J., Zhang J.-L., Wang C.-M. (2009). Overexpression of the *Arabidopsis* H+-PPase Enhanced Resistance to Salt and Drought Stress in Transgenic Alfalfa (*Medicago sativa* L.). Plant Sci..

[183] Li Z., Baldwin C.M., Hu Q., Liu H., Luo H. (2010). Heterologous Expression of *Arabidopsis* H+-Pyrophosphatase Enhances Salt Tolerance in Transgenic Creeping Bentgrass (*Agrostis stolonifera* L.). Plant Cell Environ..

[184] Pasapula V., Shen G., Kuppu S., Paez-Valencia J., Mendoza M., Hou P., Chen J., Qiu X., Zhu L., Zhang X. (2011). Expression of an *Arabidopsis* Vacuolar H+-Pyrophosphatase Gene (AVP1) in Cotton Improves Drought- and Salt Tolerance and Increases Fibre Yield in the Field Conditions. Plant Biotechnol. J..

[185] Schilling R.K., Marschner P., Shavrukov Y., Berger B., Tester M., Roy S.J., Plett D.C. (2014). Expression of the *Arabidopsis* Vacuolar H^+^-Pyrophosphatase Gene (AVP1) Improves the Shoot Biomass of Transgenic Barley and Increases Grain Yield in a Saline Field. Plant Biotechnol. J..

[186] Halder K., Chaudhuri A., Abdin M.Z., Majee M., Datta A. (2022). RNA Interference for Improving Disease Resistance in Plants and Its Relevance in This Clustered Regularly Interspaced Short Palindromic Repeats-Dominated Era in Terms of dsRNA-Based Biopesticides. Front. Plant Sci..

[187] Manzoor S., Nabi S.U., Rather T.R., Gani G., Mir Z.A., Wani A.W., Ali S., Tyagi A., Manzar N. (2024). Advancing Crop Disease Resistance through Genome Editing: A Promising Approach for Enhancing Agricultural Production. Front. Genome Ed..

[188] Song X., Dang C., Wang F., Yao H., Song Q., Ye G. (2026). CRISPR/Cas Gene Editing for Plant Resistance to Pests, Diseases, and Herbicide: Application, Risk Assessment, and Safety Evaluation. GM Crops Food.

[189] Wang D., Yang R., Liu M., Li H., Li H., Yuan W., Zhang H. (2025). Recent Advances in Innovative Strategies for Plant Disease Resistance Breeding. Front. Plant Sci..

[190] Bravo A., Gómez I., Porta H., García-Gómez B.I., Rodriguez-Almazan C., Pardo L., Soberón M. (2013). Evolution of *Bacillus Thuringiensis* Cry Toxins Insecticidal Activity. Microb. Biotechnol..

[191] Gupta M., Kumar H., Kaur S. (2021). Vegetative Insecticidal Protein (Vip): A Potential Contender from *Bacillus Thuringiensis* for Efficient Management of Various Detrimental Agricultural Pests. Front. Microbiol..

[192] Cui J., Luo J., Van Der Werf W., Ma Y., Xia J. (2011). Effect of Pyramiding Bt and CpTI Genes on Resistance of Cotton to *Helicoverpa Armigera* (Lepidoptera: *Noctuidae*) under Laboratory and Field Conditions. J. Econ. Entomol..

[193] Mercado J.A., Barceló M., Pliego C., Rey M., Caballero J.L., Muñoz-Blanco J., Ruano-Rosa D., López-Herrera C., de Los Santos B., Romero-Muñoz F. (2015). Expression of the β-1,3-Glucanase Gene Bgn13.1 from *Trichoderma Harzianum* in Strawberry Increases Tolerance to Crown Rot Diseases but Interferes with Plant Growth. Transgenic Res..

[194] Backer R., Naidoo S., van den Berg N. (2019). The NONEXPRESSOR OF PATHOGENESIS-RELATED GENES 1 (NPR1) and Related Family: Mechanistic Insights in Plant Disease Resistance. Front. Plant Sci..

[195] Osman G.H., Assem S.K., Alreedy R.M., El-Ghareeb D.K., Basry M.A., Rastogi A., Kalaji H.M. (2015). Development of Insect Resistant Maize Plants Expressing a Chitinase Gene from the Cotton Leaf Worm, *Spodoptera littoralis*. Sci. Rep..

[196] Corrado G., Arciello S., Fanti P., Fiandra L., Garonna A., Digilio M.C., Lorito M., Giordana B., Pennacchio F., Rao R. (2008). The Chitinase A from the Baculovirus AcMNPV Enhances Resistance to Both Fungi and Herbivorous Pests in Tobacco. Transgenic Res..

[197] Wu W., Chen N., Hu J., Zhang J., Wu Y., Yao T., Wang X. (2025). Function of Chitinase in *Penicillium Ochrochloron* Q-3-1 and Its Insecticidal and Antifungal Mechanism and Biological Control against Citrus Pests and Diseases. Int. J. Biol. Macromol..

[198] Sabir M., Anwar Y., Khan A., Ali M., Yousuf P.Y., Al-Ghamdi K., Hakeem K.R. (2016). ChiC Gene Enhances Fungal Resistance in Indigenous Potato Variety (Diamant) Via *Agrobacterium*-Mediated Transformation. Biosci. Biotech. Res. Asia.

[199] Kumar S., Behera L., Kumari R., Bag D., Sowmya V., Keswani C., Minkina T., Bouket A.C., Dutta P., Nehela Y. (2024). Unraveling the Role of Antimicrobial Peptides in Plant Resistance against Phytopathogens. Discov. Sustain..

[200] Kim S.H., Min Y.-H., Park M.C. (2025). Antimicrobial Peptides: Current Status, Mechanisms of Action, and Strategies to Overcome Therapeutic Limitations. Microorganisms.

[201] Coca M., Peñas G., Gómez J., Campo S., Bortolotti C., Messeguer J., Segundo B.S. (2006). Enhanced Resistance to the Rice Blast Fungus *Magnaporthe Grisea* Conferred by Expression of a Cecropin a Gene in Transgenic Rice. Planta.

[202] Gruden Š., Poklar Ulrih N. (2021). Diverse Mechanisms of Antimicrobial Activities of Lactoferrins, Lactoferricins, and Other Lactoferrin-Derived Peptides. Int. J. Mol. Sci..

[203] Stefanova G., Slavov S., Gecheff K., Vlahova M., Atanassov A. (2013). Expression of Recombinant Human Lactoferrin in Transgenic Alfalfa Plants. Biol. Plant..

[204] Buziashvili A., Yemets A. (2023). Lactoferrin and Its Role in Biotechnological Strategies for Plant Defense against Pathogens. Transgenic Res..

[205] Liu K., Tong Z., Zhang X., Dahmani M., Zhao M., Hu M., Li X., Xue Z. (2024). A Review: Development of a Synthetic Lactoferrin Biological System. BioDesign Res..

[206] Reinders J.D., Reinders E.E., Robinson E.A., French B.W., Meinke L.J. (2022). Evidence of Western Corn Rootworm (*Diabrotica Virgifera* Virgifera LeConte) Field-Evolved Resistance to Cry3Bb1 + Cry34/35Ab1 Maize in Nebraska. Pest. Manag. Sci..

[207] Liu L., Gao M., Yang S., Liu S., Wu Y., Carrière Y., Yang Y. (2017). Resistance to *Bacillus Thuringiensis* Toxin Cry2Ab and Survival on Single-Toxin and Pyramided Cotton in Cotton Bollworm from China. Evol. Appl..

[208] Santos-Amaya O.F., Rodrigues J.V.C., Souza T.C., Tavares C.S., Campos S.O., Guedes R.N.C., Pereira E.J.G. (2015). Resistance to Dual-Gene Bt Maize in *Spodoptera Frugiperda*: Selection, Inheritance and Cross-Resistance to Other Transgenic Events. Sci. Rep..

[209] Clemente M., Corigliano M.G., Pariani S.A., Sánchez-López E.F., Sander V.A., Ramos-Duarte V.A. (2019). Plant Serine Protease Inhibitors: Biotechnology Application in Agriculture and Molecular Farming. Int. J. Mol. Sci..

[210] Khadeeva N.V., Kochieva E.Z., Tcherednitchenko M.Y., Yakovleva E.Y., Sydoruk K.V., Bogush V.G., Dunaevsky Y.E., Belozersky M.A. (2009). Use of Buckwheat Seed Protease Inhibitor Gene for Improvement of Tobacco and Potato Plant Resistance to Biotic Stress. Biochemistry.

[211] Yasuda N., Mitsunaga T., Hayashi K., Koizumi S., Fujita Y. (2015). Effects of Pyramiding Quantitative Resistance Genes Pi21, Pi34, and Pi35 on Rice Leaf Blast Disease. Plant Dis..

[212] Zhang Y., Zhao J., Li Y., Yuan Z., He H., Yang H., Qu H., Ma C., Qu S. (2016). Transcriptome Analysis Highlights Defense and Signaling Pathways Mediated by Rice Pi21 Gene with Partial Resistance to *Magnaporthe oryzae*. Front. Plant Sci..

[213] Nawaz G., Usman B., Peng H., Zhao N., Yuan R., Liu Y., Li R. (2020). Knockout of Pi21 by CRISPR/Cas9 and iTRAQ-Based Proteomic Analysis of Mutants Revealed New Insights into *M*. *Oryzae* Resistance in Elite Rice Line. Genes.

[214] Rajam M.V., Chandola N., Saiprasad Goud P., Singh D., Kashyap V., Choudhary M.L., Sihachakr D. (2007). Thaumatin Gene Confers Resistance to Fungal Pathogens as Well as Tolerance to Abiotic Stresses in Transgenic Tobacco Plants. Biol. Plant.

[215] Keil R., De Oliveira Neves L., Da Silva L.C.O., Lamb T.I., Berghahn E., Pita F.M., Johann L., Wang Y., Feng Z., Wang G. (2024). Osmotin1 Is Involved in Rice Resistance to *Schizotetranychus oryzae* (Acari: *Tetranychidae*) Infestation. Pest. Manag. Sci..

[216] Bhattacharya A., Saini U., Joshi R., Kaur D., Pal A.K., Kumar N., Gulati A., Mohanpuria P., Yadav S.K., Kumar S. (2014). Osmotin-Expressing Transgenic Tea Plants Have Improved Stress Tolerance and Are of Higher Quality. Transgenic Res..

[217] Hakim, Ullah A., Hussain A., Shaban M., Khan A.H., Alariqi M., Gul S., Jun Z., Lin S., Li J. (2018). Osmotin: A Plant Defense Tool against Biotic and Abiotic Stresses. Plant Physiol. Biochem..

[218] Yuan L., Gai W., Xuan X., Ahiakpa J.K., Li F., Ge P., Zhang X., Tao J., Yang Y., Zhang Y. (2025). Advances in Improving Tomato Fruit Quality by Gene Editing. Hortic. Plant J..

[219] Nguyen H.C., Hoefgen R., Hesse H. (2012). Improving the Nutritive Value of Rice Seeds: Elevation of Cysteine and Methionine Contents in Rice Plants by Ectopic Expression of a Bacterial Serine Acetyltransferase. J. Exp. Bot..

[220] Jiang S.-Y., Ma A., Xie L., Ramachandran S. (2016). Improving Protein Content and Quality by Over-Expressing Artificially Synthetic Fusion Proteins with High Lysine and Threonine Constituent in Rice Plants. Sci. Rep..

[221] Swamy B.P.M., Rahman M.A., Inabangan-Asilo M.A., Amparado A., Manito C., Chadha-Mohanty P., Reinke R., Slamet-Loedin I.H. (2016). Advances in Breeding for High Grain Zinc in Rice. Rice.

[222] Das P., Adak S., Lahiri Majumder A. (2020). Genetic Manipulation for Improved Nutritional Quality in Rice. Front. Genet..

[223] Sidahmed H., Vad A., Nagy J. (2025). Advances in Sweet Corn (*Zea mays* L. *Saccharata*) Research from 2010 to 2025: Genetics, Agronomy, and Sustainable Production. Agronomy.

[224] Ye X., Al-Babili S., Klöti A., Zhang J., Lucca P., Beyer P., Potrykus I. (2000). Engineering the Provitamin A (Beta-Carotene) Biosynthetic Pathway into (Carotenoid-Free) Rice Endosperm. Science.

[225] Paine J.A., Shipton C.A., Chaggar S., Howells R.M., Kennedy M.J., Vernon G., Wright S.Y., Hinchliffe E., Adams J.L., Silverstone A.L. (2005). Improving the Nutritional Value of Golden Rice through Increased Pro-Vitamin A Content. Nat. Biotechnol..

[226] Huang J., Liu F., Zhang J., Tang B., Deng J., Shi T., Zhu L., Li H., Chen Q. (2025). Identification of the Granule-Bound Starch Synthase (GBSS) Genes Involved in Amylose Biosynthesis in Tartary Buckwheat (*Fagopyrum tataricum* (L.) Gaertn.). Plants.

[227] Fujita N., Miura S., Crofts N. (2022). Effects of Various Allelic Combinations of Starch Biosynthetic Genes on the Properties of Endosperm Starch in Rice. Rice.

[228] Jayarathna S., Hofvander P., Péter-Szabó Z., Andersson M., Andersson R. (2024). GBSS Mutations in an SBE Mutated Background Restore the Potato Starch Granule Morphology and Produce Ordered Granules despite Differences to Native Molecular Structure. Carbohydr. Polym..

[229] Cui W., Zhuang Z., Jiang P., Pan J., Zhao G., Xu S., Shen W. (2022). Characterization, Expression Profiling, and Biochemical Analyses of the Cinnamoyl-CoA Reductase Gene Family for Lignin Synthesis in Alfalfa Plants. Int. J. Mol. Sci..

[230] De Meester B., Madariaga Calderón B., De Vries L., Pollier J., Goeminne G., Van Doorsselaere J., Chen M., Ralph J., Vanholme R., Boerjan W. (2020). Tailoring Poplar Lignin without Yield Penalty by Combining a Null and Haploinsufficient CINNAMOYL-CoA REDUCTASE2 Allele. Nat. Commun..

[231] Wani A.B., Noor W., Pandit A., Husaini A.M. (2025). Upregulated Expression of MYB4, DREB1 and AP37 Transcription Factors Modulates Cold Stress Response in High-Altitude Himalayan Rice via Time-Dependent ROS Regulation. Mol. Biol. Rep..

[232] Jacob P., Hirt H., Bendahmane A. (2017). The Heat-Shock Protein/Chaperone Network and Multiple Stress Resistance. Plant Biotechnol. J..

[233] Li J., Meng L., Ren S., Jia C., Liu R., Jiang H., Chen J. (2023). OsGSTU17, a Tau Class Glutathione S-Transferase Gene, Positively Regulates Drought Stress Tolerance in *Oryza sativa*. Plants.

[234] Wahyudi A., Fukazawa C., Motohashi R. (2020). Function of SlTILs and SlCHL under Heat and Oxidative Stresses in Tomato. Plant Biotechnol..

[235] Abdul Aziz M., Brini F., Jrad O., Rahman S., Ahmad M., Vijayan R., Masmoudi K. (2025). Molecular Propensity and Stress Tolerance of Dehydrins from Desert Plants. Sci. Rep..

[236] Cui X., Cao Y., Lv M., Zhou S., Chen M., Li C., Zhang H. (2025). The Translation Initiation Factor eIF2 Is Phosphorylated to Inhibit Protein Translation through Reactive Oxygen Species under Nutrient Deficiencies in *Arabidopsis*. Stress. Biol..

[237] Zhang H., Wu T., Li Z., Huang K., Kim N.-E., Ma Z., Kwon S.-W., Jiang W., Du X. (2021). OsGATA16, a GATA Transcription Factor, Confers Cold Tolerance by Repressing OsWRKY45-1 at the Seedling Stage in Rice. Rice.

[238] Park M.-R., Yun K.-Y., Mohanty B., Herath V., Xu F., Wijaya E., Bajic V.B., Yun S.-J., De Los Reyes B.G. (2010). Supra-Optimal Expression of the Cold-Regulated OsMyb4 Transcription Factor in Transgenic Rice Changes the Complexity of Transcriptional Network with Major Effects on Stress Tolerance and Panicle Development. Plant Cell Environ..

[239] Laura M., Consonni R., Locatelli F., Fumagalli E., Allavena A., Coraggio I., Mattana M. (2010). Metabolic Response to Cold and Freezing of *Osteospermum Ecklonis* Overexpressing Osmyb4. Plant Physiol. Biochem..

[240] Pasquali G., Biricolti S., Locatelli F., Baldoni E., Mattana M. (2008). Osmyb4 Expression Improves Adaptive Responses to Drought and Cold Stress in Transgenic Apples. Plant Cell Rep..

[241] Rai A.C., Singh I., Singh M., Shah K. (2014). Expression of ZAT12 Transcripts in Transgenic Tomato under Various Abiotic Stresses and Modeling of ZAT12 Protein in Silico. Biometals.

[242] Rai A.C., Singh M., Shah K. (2013). Engineering Drought Tolerant Tomato Plants Over-Expressing BcZAT12 Gene Encoding a C_2_H_2_ Zinc Finger Transcription Factor. Phytochemistry.

[243] Shah K., Singh M., Rai A.C. (2013). Effect of Heat-Shock Induced Oxidative Stress Is Suppressed in BcZAT12 Expressing Drought Tolerant Tomato. Phytochemistry.

[244] Wang L., Wang S., Tong R., Wang S., Yao J., Jiao J., Wan R., Wang M., Shi J., Zheng X. (2022). Overexpression of PgCBF3 and PgCBF7 Transcription Factors from Pomegranate Enhances Freezing Tolerance in *Arabidopsis* under the Promoter Activity Positively Regulated by PgICE1. Int. J. Mol. Sci..

[245] Zhou M., Chen H., Wei D., Ma H., Lin J. (2017). *Arabidopsis* CBF3 and DELLAs Positively Regulate Each Other in Response to Low Temperature. Sci. Rep..

[246] Zhang G., Wang J., Yu M., Bai M., Chen M., Yang L., Wu Z., Gu C. (2025). Transcription Regulation Network Revealed the Role of CBF3 Gene in Response to Low-Temperature Stress of *Cuphea hookeriana*. Ind. Crops Prod..

[247] Chen M., Xu Z., Xia L., Li L., Cheng X., Dong J., Wang Q., Ma Y. (2009). Cold-Induced Modulation and Functional Analyses of the DRE-Binding Transcription Factor Gene, GmDREB3, in Soybean (*Glycine max* L.). J. Exp. Bot..

[248] Bai X., Zhou Y., Islam M.A., Zhang W., Ning L., Ling B., Wang Y., Xu Z., Sun D., Chen M. (2022). A Soybean *GmDREB3* Gene Contributes to Drought Tolerance in Wheat. Food Energy Secur..

[249] Noack F., Engist D., Gantois J., Gaur V., Hyjazie B.F., Larsen A., M’Gonigle L.K., Missirian A., Qaim M., Sargent R.D. (2024). Environmental Impacts of Genetically Modified Crops. Science.

[250] Giraldo P.A., Shinozuka H., Spangenberg G.C., Cogan N.O.I., Smith K.F. (2019). Safety Assessment of Genetically Modified Feed: Is There Any Difference from Food?. Front. Plant Sci..

[251] Karthikeyan A., Dhali A.M., Jain P., Al-Khayri J.M., Yatoo A.M., Jain S.M., Penna S. (2026). Environmental and Health Risk Assessment of GM Crops. Handbook of Agricultural Technologies.

[252] Delaney A., Evans T., McGreevy J., Blekking J., Schlachter T., Korhonen-Kurki K., Tamás P.A., Crane T.A., Eakin H., Förch W. (2018). Governance of Food Systems across Scales in Times of Social-Ecological Change: A Review of Indicators. Food Sec..

[253] Poulsen M., Schrøder M., Wilcks A., Kroghsbo S., Lindecrona R.H., Miller A., Frenzel T., Danier J., Rychlik M., Shu Q. (2007). Safety Testing of GM-Rice Expressing PHA-E Lectin Using a New Animal Test Design. Food Chem. Toxicol..

[254] Hajimohammadi B., Eslami G., Zandi H., Ehrampoush M.H., Naimi A., Derakhshan M., Hedayat P., Fallahi R., Fallahzadeh H., Rezvani M.E. (2021). Safety Assessment of Genetically Modified Rice Expressing Cry1Ab Protein in Sprague–Dawley Rats. Sci. Rep..

[255] Bhatti F., Asad S., Khan Q.M., Mobeen A., Iqbal M.J., Asif M. (2019). Risk Assessment of Genetically Modified Sugarcane Expressing AVP1 Gene. Food Chem. Toxicol..

[256] Carlson A.B., Mukerji P., Mathesius C.A., Huang E., Herman R.A., Hoban D., Thurman J.D., Roper J.M. (2020). DP-2Ø2216-6 Maize Does Not Adversely Affect Rats in a 90-Day Feeding Study. Regul. Toxicol. Pharmacol..

[257] Smith B.L., Carlson A.B., Fallers M.N., Crumplar S.S., Zimmermann C.S., Mathesius C.A., Mukerji P., McNaughton J.L., Herman R.A. (2024). Rodent and Broiler Feeding Studies with Maize Containing Genetically Modified Event DP-915635-4 Show No Adverse Effects on Health or Performance. Food Chem. Toxicol..

[258] Chen C., Shi L., Mao H., Han C., Zhao J., Zhuo Q., Li Y. (2024). Safety Assessment of Transgenic Maize CC-2 by 90-Day Feeding Study in Sprague-Dawley Rats. Toxicol. Res..

[259] Liu A., Yin X., Wen J., Hou C., Zhou R., Liu X., Yin N., Jian Y., Liu S., Zhang X. (2025). Relative Safety of Glyphosate-Resistant Maize (CC-2) in Rats. GM Crops Food.

[260] Mullins E., Bresson J., Dalmay T., Dewhurst I.C., Epstein M.M., Firbank L.G., Guerche P., Hejatko J., Naegeli H., EFSA Panel on Genetically Modified Organisms (GMO) (2021). Assessment of Genetically Modified Maize NK603 × T25 × DAS-40278-9 and Subcombinations, for Food and Feed Uses, under Regulation (EC) No 1829/2003 (Application EFSA-GMO-NL-2019-164). EFSA J..

[261] Lin H.-T., Lee W.-C., Tsai Y.-T., Wu J.-H., Yen G.-C., Yeh S.-D., Cheng Y.-H., Chang S.-C., Liao J.-W. (2016). Subchronic Immunotoxicity Assessment of Genetically Modified Virus-Resistant Papaya in Rats. J. Agric. Food Chem..

[262] Wang X., He X., Zou S., Xu W., Jia X., Zhao B., Zhao C., Huang K., Liang Z. (2016). A Subchronic Feeding Study of Dicamba-Tolerant Soybean with the Dmo Gene in Sprague-Dawley Rats. Regul. Toxicol. Pharmacol..

[263] Magaña-Gómez J.A., Calderón De La Barca A.M. (2009). Risk Assessment of Genetically Modified Crops for Nutrition and Health. Nutr. Rev..

[264] Zhou Q., Liu Y., Zhang S., Li S., Zhao M., Zhou X., Zhou D., Qian Z. (2025). Food Safety Assessment of Genetically Modified Soybean DBN9004×DBN8002×DBN8205 in a Subchronic Rodent Feeding Study. Food Chem. Toxicol..

[265] Chowdhury E.H., Kuribara H., Hino A., Sultana P., Mikami O., Shimada N., Guruge K.S., Saito M., Nakajima Y. (2003). Detection of Corn Intrinsic and Recombinant DNA Fragments and Cry1Ab Protein in the Gastrointestinal Contents of Pigs Fed Genetically Modified Corn Bt11 1. J. Anim. Sci..

[266] Hammond B.G., Jez J.M. (2011). Impact of Food Processing on the Safety Assessment for Proteins Introduced into Biotechnology-Derived Soybean and Corn Crops. Food Chem. Toxicol..

[267] Hu Y., Guo M., Zhuo Q., Han C., Shi L., Mao H., Li Y., Zhao J., Chen C., Yang X. (2020). Three-Generation Reproductive Toxicity of Genetically Modified Maize with *Cry1Ab* and *Epsps* Genes in Rats. J. Agric. Food Chem..

[268] Tian J., Ke X., Yuan Y., Yang W., Tang X., Qu J., Qu W., Fu S., Zheng Y., Fan J. (2021). Two Generation Reproduction Toxicity Study of GmDREB3 Gene Modified Wheat in Wistar Rats. Food Chem. Toxicol..

[269] Domingo J.L. (2025). Genetically Modified Crops: Balancing Safety, Sustainability, and Global Security. Environ. Res..

[270] Reynier E., Rubin E. (2025). Glyphosate Exposure and GM Seed Rollout Unequally Reduced Perinatal Health. Proc. Natl. Acad. Sci. USA.

[271] Arregui M.C., Lenardón A., Sanchez D., Maitre M.I., Scotta R., Enrique S. (2004). Monitoring Glyphosate Residues in Transgenic Glyphosate-Resistant Soybean. Pest. Manag. Sci..

[272] Duke S.O., Rimando A.M., Pace P.F., Reddy K.N., Smeda R.J. (2003). Isoflavone, Glyphosate, and Aminomethylphosphonic Acid Levels in Seeds of Glyphosate-Treated, Glyphosate-Resistant Soybean. J. Agric. Food Chem..

[273] Duke S.O., Carvalho L.B. (2025). Unintended Effects of the Intended Herbicides on Transgenic Herbicide-Resistant Crops. Agronomy.

[274] Caradus J.R. (2022). Intended and Unintended Consequences of Genetically Modified Crops—Myth, Fact and/or Manageable Outcomes?. New Zealand J. Agric. Res..

[275] Acquavella J.F., Alexander B.H., Mandel J.S., Burns C.J., Gustin C. (2006). Exposure Misclassification in Studies of Agricultural Pesticides: Insights from Biomonitoring. Epidemiology.

[276] Dias M., Rocha R., Soares R.R. (2023). Down the River: Glyphosate Use in Agriculture and Birth Outcomes of Surrounding Populations. Rev. Econ. Stud..

[277] Skidmore M.E., Sims K.M., Gibbs H.K. (2023). Agricultural Intensification and Childhood Cancer in Brazil. Proc. Natl. Acad. Sci. USA.

[278] Ahmed A.U., Hoddinott J., Abedin N., Hossain N. (2021). The Impacts of GM Foods: Results from a Randomized Controlled Trial of Bt Eggplant in Bangladesh. Am. J. Agric. Econ..

[279] Veettil P.C., Krishna V.V., Qaim M. (2017). Ecosystem Impacts of Pesticide Reductions through Bt Cotton Adoption. Aust. J. Agric. Resour. Econ..

[280] Gassmann A.J., Reisig D.D. (2023). Management of Insect Pests with Bt Crops in the United States. Annu. Rev. Entomol..

[281] Bhardwaj V., Bhardwaj N., Kumari P., Al-Khayri J.M., Yatoo A.M., Jain S.M., Penna S. (2025). Genetically Modified Crops for Pesticide Use Reduction. Handbook of Agricultural Technologies.

[282] Ngongolo K., Mmbando G.S. (2025). Necessities, Environmental Impact, and Ecological Sustainability of Genetically Modified (GM) Crops. Discov. Agric..

[283] Rastogi Verma S. (2013). Genetically Modified Plants: Public and Scientific Perceptions. ISRN Biotechnol..

[284] Nordlee J.A., Taylor S.L., Townsend J.A., Thomas L.A., Bush R.K. (1996). Identification of a Brazil-Nut Allergen in Transgenic Soybeans. N. Engl. J. Med..

[285] Lee S., Moschini G., Perry E.D. (2023). Genetically Engineered Varieties and Applied Pesticide Toxicity in U.S. Maize and Soybeans: Heterogeneous and Evolving Impacts. Ecol. Econ..

[286] Dainese M., Martin E.A., Aizen M.A., Albrecht M., Bartomeus I., Bommarco R., Carvalheiro L.G., Chaplin-Kramer R., Gagic V., Garibaldi L.A. (2019). A Global Synthesis Reveals Biodiversity-Mediated Benefits for Crop Production. Sci. Adv..

